# The effect of treatment with a non-ionic surfactant vesicular formulation of sodium stibogluconate on host immune responses and serum metabolites in a murine model of *Leishmania donovani*

**DOI:** 10.3389/fimmu.2025.1499513

**Published:** 2025-10-02

**Authors:** Raphael Taiwo Aruleba, Bernard Ong’ondo Osero, Du Toit Loots, Laneke Luies, Zama Cele, Priscilla Abena Ankamaa Opare, Mari van Reenen, Frank Brombacher, Katharine C. Carter, Ramona Hurdayal

**Affiliations:** ^1^ Department of Molecular and Cell Biology, University of Cape Town, Cape Town, South Africa; ^2^ Division of Immunology, Department of Pathology, Faculty of Health Sciences, Institute of Infectious Diseases and Molecular Medicine (IDM), South African Medical Research Council (SAMRC) on Immunology of Infectious Diseases, University of Cape Town, Cape Town, South Africa; ^3^ Centre for Biotechnology Research and Development, Kenya Medical Research Institute, Nairobi, Kenya; ^4^ Faculty of Health Sciences, Wellcome Centre for Infectious Diseases Research in Africa, Institute of Infectious Diseases and Molecular Medicine (IDM), University of Cape Town, Cape Town, South Africa; ^5^ Focus Area Human Metabolomics, North-West University, Potchefstroom, South Africa; ^6^ Strathclyde Institute of Pharmacy and Biomedical Sciences, University of Strathclyde, Glasgow, United Kingdom

**Keywords:** *Leishmania donovani*, chemotherapy, immunity, metabolomics, mouse

## Abstract

**Introduction:**

Visceral leishmaniasis (VL), caused by *Leishmania donovani*, is associated with parasite-induced immunological and physiological changes that ensure the survival of amastigotes within the host. Both the parasite and the host have nutritional requirements, and for auxotrophic *Leishmania*, dependence on the host to supply specific growth requirements is essential. This highlights an intricate link between host immunity and metabolism during VL. This study explores the interplay between the host metabolome and immune responses pre- and post-infection and treatment, aiming to identify early metabolite markers of therapeutic success against *Leishmania*.

**Methods:**

BALB/c mice infected with *L. donovani* were divided into cured and non-cured groups based on treatment with a non-ionic surfactant vesicle formulation of sodium stibogluconate (300 mg Sb^v^/kg, SSG-NIV) or PBS vehicle control. Specific immune responses were determined at day 21 and day 60 post-infection, and serum metabolite levels was measured using untargeted GC×GC-TOFMS metabolomics.

**Results and discussions:**

Treatment effectively reduced parasite loads, triggering heightened CD4+ and CD8+ T-cell responses at day 21, with increased IFN-γ, IL-12, and IL-4, and decreased IL-10 and TGF-β. Pre-treatment metabolomics analysis identified changes in glycolysis, fatty acid and amino acid metabolism 1-week PI, suggesting an increased Warburg effect to supplement parasite survival and initiation of immune responses. Valine, lactic acid, and glycerol-1-oleate were identified as markers of early infection. Treatment with SSG-NIV altered metabolism of major macromolecules and the TCA cycle relative to non-cured groups. Additionally, glycine and ribitol show promise as immune correlates for antiparasitic therapies. These findings highlight the diagnostic and prognostic potential of serum-derived metabolites in monitoring host immune responses to VL and treatment.

## Graphical Abstract

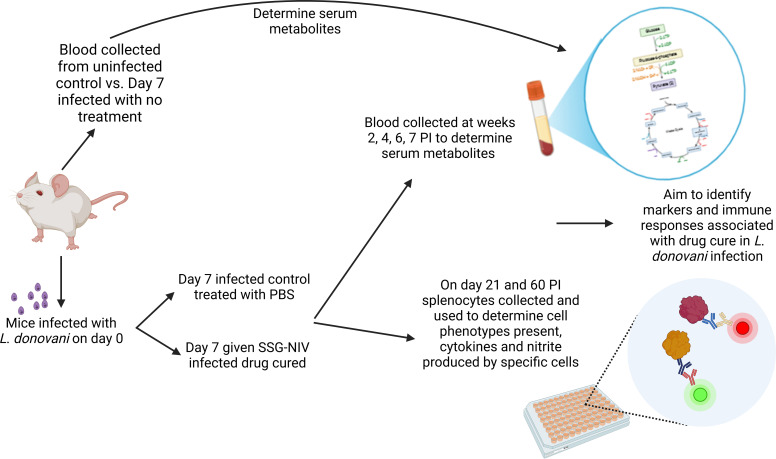

## 1 Introduction

Visceral leishmaniasis (VL), also known as kala-azar, is a life-threatening vector-borne disease caused by infection with the intracellular protozoan parasites *L. donovani* or *L*. *infantum*. This neglected tropical disease primarily affects populations in developing countries, with approximately 50,000 to 90,000 new cases reported annually worldwide (1). Despite ongoing efforts to control the disease, VL remains a significant public health concern due to its high morbidity and mortality rates. The limited number of drugs available to treat VL, most of which require patient hospitalization and have been in use for over three decades, present various problems and cause negative effects, hence limiting their clinical use (2).

The nature, genetic makeup, and immune status of the host, along with the features of the parasite, are determinants of the severity and clinical outcome (3). The ability to mount a robust host immune response plays a critical role in the successful elimination of VL. Both innate and adaptive arms of the immune system orchestrate the complex interplay between the host and the parasite, leading to either parasite clearance or disease progression (4, 5). The parasites invade and reside within macrophages, which are crucial to the host defense mechanisms. Although traditional immunological studies have provided valuable insights into the host immune response against *Leishmania*, the underlying molecular mechanisms governing this intricate interplay are not yet fully understood.

Understanding host metabolism will assist in recognizing the energy sources and building blocks the parasite exploits in the host. For example, infections like malaria alter metabolic activities in mice, resulting in parasite-induced hepatosplenomegaly (6). Hepatosplenomegaly a symptom of liver disease, is associated with metabolic disorders (7). Given that hepatosplenomegaly is one of the main clinical symptoms of VL infection, it would not be surprising if metabolic functions are dysregulated in the host. Moreover, *Leishmania* parasites can alter lipid metabolism, potentially revealing further metabolic dysfunction. To gain a deeper understanding of the immunometabolism of VL during infection and drug treatment, integrating immunology with metabolomics is crucial. This integration can unveil the metabolic signatures and pathways that underpin the host-parasite interaction and may also give insights into the management of conditions with similar clinical features upon successful drug treatment.

Metabolomics, a rapidly evolving field of systems biology, holds immense potential for deciphering the intricate metabolic alterations associated with disease progression and host-pathogen interactions (8). As VL is an immune-driven disease, studying the metabolic profiles of the host is crucial for early diagnosis, guiding treatment decisions, and preventing relapse. This highlights the importance of understanding the relationship between immune response, host metabolism, and parasite survival in VL. Importantly, studying the host pre- and post-drug administration can additionally aid in monitoring treatment response, ultimately improving treatment outcomes. However, most studies have primarily focused on parasite-drug interactions, with limited data on metabolite profiles in the host during VL following infection and treatment.

Consequently, there is an urgent need to identify distinct, sensitive, and specific markers that can enhance diagnosis and serve as surrogate endpoints to monitor treatment response, before completing a treatment course. Indeed, early diagnosis of VL is crucial for better treatment outcomes and reduced relapses. Metabolomic markers offer potential improvements over invasive diagnostic methods like splenic aspirates. Given the socioeconomic context of leishmaniasis, diagnostic tools should be non-invasive, cost-effective, easily accessible and easy to use at the point-of-care (9). In this regard, serum metabolites can offer insights into disease status and host responses, making them ideal for point-of-care testing. Serum metabolomics has proven valuable in biomarker discovery for various disorders, with GC×GC-TOFMS being particularly well-suited for metabolic profiling of serum samples due to its reproducibility and sensitivity.

Herein, we show the intricate metabolic alterations associated with immune responses to murine VL-treated with sodium stibogluconate delivered in non-ionic surfactant vesicles (SSG-NIV), shedding light on the potential underlying molecular mechanisms of host-parasite interactions. Such insights may pave the way for the development of innovative diagnostic tools, targeted therapies, and biomarkers that can aid the clinical management of VL. A graphical overview of the study design is presented in the graphical abstract.

## 2 Materials and methods

### 2.1 Ethical statement

This study followed the recommendations of the South African national guidelines and the University of Cape Town (UCT) laboratory animal procedures guidelines. All animal experiments had ethical approval from the Faculty of Health Sciences Animal Ethics Committee (FHS AEC) (FHS AEC: 018/034).

### 2.2 Animals and parasites

This study utilized age and sex-matched BALB/c mice (8–10 weeks, both male and female, n = 20 for each experiment) supplied from the UCT colony. Mice were intravenously injected via the tail vein, without anesthesia, with 2 × 10^7^ *L. donovani* amastigotes (MHOM/ET/67:LV82), obtained from the spleen of Rag1^-/-^ mice. On day 7 post-infection (PI), mice were divided into two groups (n = 10/group). One group received a single dose of SSG-NIV (300 mg of Sb^v^/kg, drug-cured group), a dose previously shown to significantly reduce parasite burdens (10, 11). The SSG-NIV was prepared from a lyophilized empty surfactant NIV formulation, hydrated with SSG solution (100 mg/ml, 29.94% wt/wt Sb^V^) immediately before use. The second group was treated with a single dose of PBS (infected control group). We compared the response of SSG-NIV and empty-NIV in a previous study (12). Treatment with empty-formulation had no significant effect on parasite burdens or host immune responses compared to infected controls i.e. specific IgG1 and IgG2a endpoint titres and proliferative responses of splenocytes to specific antigen. Accordingly, this treatment group was not included in this study (12). Serum samples were collected from each mouse and immediately frozen for metabolite profiling at various time points from week 1 post-infection (PI) and weeks 2, 4, 6, and 7 post-treatment (PT). On days 21 and 60, five mice from each group were sacrificed, and spleen, liver, serum, and bone marrow samples were collected for further immunological analysis.

### 2.3 Determination of parasite burden

Parasite burdens in the liver, spleen, and bone marrow of each mouse were determined using the method described by (10). Leishman-Donovan units (LDU) were determined as the number of amastigotes per 1000 host cell nuclei × organ weight (g).

### 2.4 Isolation and *ex-vivo* stimulation of spleen cells

The spleen of each mouse was removed at sacrifice and processed as described in previous studies (13). Spleen cells were seeded at 1 × 10^6^ cells per well and incubated at 37^0^C with 5% CO_2_ for 72 hours in the presence of anti-(α)-CD3 [20 µg/ml] or LPS [10 ng/ml]. The concentrations of IFN-γ, IL-12, IL-4, IL-10, and TGF-β were detected in the cell supernatants and serum via ELISA as previously described (14). Nitrite levels and arginase activity were measured in the supernatants of LPS-stimulated splenocytes as detailed in previous studies (15).

### 2.5 Flow cytometry

Spleen cells were seeded in a 96-well v-bottom plate at a concentration of 1 × 10^6^ cells per well (16). The plate was then centrifuged at 300 g for 5 minutes at 4°C. The cell pellet was resuspended in 50 µl of antibody surface mix containing 1% v/v rat serum and 1% v/v FC-γ blocker (clone 2.4G2) and incubated for 30 minutes in the dark. After washing with FACS buffer, the plate was centrifuged at 300 g for 5 minutes at 4°C to remove unbound antibodies and then resuspended in 200 µL FACS buffer for acquisition. Specific surface markers for T cells, B cells, macrophages, and dendritic cells (DCs) were detected using monoclonal antibodies conjugated to specific fluorochromes. The T and B cell panels included markers such as CD3, CD19, CD44, CD62L, and CD8. The macrophages and DCs panel included markers such as CD11c, CD11b, F4/80, and MHCII. Cytokine panels included markers such as IFN-γ, IL-10, and IL-4. Cell acquisition was performed using a BD LSRFortessa Machine (BD Biosciences, USA), and the obtained data were analyzed using FlowJo software version 10.5.3 (Treestar, USA).

### 2.6 Sample extraction and derivatization for metabolomics

Metabolites were extracted from serum samples (40 µL) of the SSG-NIV treated mice, control, and uninfected control mice by adding 40 µL of an internal standard solution (3-phenylbutyric acid dissolved in chloroform:methanol:water with a ratio of 1:3:1 and a final concentration of 50 ppm). Proteins were precipitated by adding 240 µL of acetonitrile, vortexing the samples at maximum speed, incubating on ice for 10 minutes, and centrifuging at 2000 g for 10 minutes at 4°C. The resulting supernatant was transferred to a GC×GC-TOFMS sample vial and dried using nitrogen gas (17). Prior to GC×GC-TOFMS analysis, all samples underwent derivatization through oximation and silylation. Oximation involved adding 22 µL methoxyamine hydrochloride dissolved in pyridine (20 mg/ml) to the dried samples, followed by incubation at 50°C for 90 minutes. Silylation was performed by adding 32 µL N,O-Bis(trimethylsilyl)trifluoroacetamide containing 1% v/v trimethylchlorosilane, with further incubation at 60°C for 60 minutes (18).

### 2.7 GCxGC-TOFMS analysis

Derivatized samples were transferred to GC-MS vials containing inserts and placed in an Agilent 7693 auto-sampler tray coupled to a GC-TOFMS system comprising a 7890 GC system and a LECO Pegasus HT mass analyzer. Each sample extract, along with quality control (QC) samples and sample blanks, was injected (1 µL) randomly using a 1:5 split ratio onto the GC×GC-TOFMS. Chromatographic separation was achieved using a Restek RXi^®^-5 (20m × 0.2mm × 0.18μm) primary column and a Restek RXi^®^-17 (1m × 0.2mm × 0.18μm) secondary column. The inlet temperature was set at 250°C, the transfer line temperature at 225°C, and the ion source temperature at 200°C throughout the run. Helium served as the carrier gas. The oven temperature was set at 50^0^C for 1 minute, and then increased to a final temperature of 300^0^C. The total run time for each sample was approximately 33 minutes, with a solvent delay of 350 s before data acquisition. Mass spectra were collected from 50–800 m/z at an acquisition rate of 20 spectra per second, with a detector voltage of 1600V and filament bias of -70eV (17).

Data were analyzed using ChromaTOF software version 4.72.0.0 (LECO, St. Joseph, MI, USA). Baseline subtraction, peak detection, and deconvolution were performed, with smoothing parameters set by the software. Peaks were identified based on criteria such as peak width of at least 3 s, a signal-to-noise ratio of 100, and a minimum of five apexing masses. Peak annotation relied on using spectral matching to the National Institute of Standards and Technology (NIST) spectral libraries (mainlib, replib), an in-house library, and sugar standards injected during the run, requiring a 60% similarity spectral match (19).

### 2.8 Data clean-up and statistical analysis

All metabolite values were normalized to the internal standard, and further clean-up steps were applied (17). Zero-filtering excluded compounds with over 50% zero-valued observations in both mice groups. The remaining zero values were replaced with random numbers from a uniform distribution. Non-parametric univariate statistics were applied to the zero-replaced data. Principle component analysis (PCA) assessed the quality of the analysis, visualizing variations. Batch correction efforts failed to resolve observed variation, leading to the exclusion of batch 1. Log transformation was applied to improve data normality. Parametric univariate statistics were performed on the log-transformed data, and Pareto scaling addressed differences in orders of magnitude. Multivariate statistics were conducted on the scaled data without removing outliers due to equal group sizes. MetaboAnalyst was used, selecting metabolites between SSG-NIV treated mice and controls based on fold change greater than or equal to 1.5, and Cohen’s d-value identified significant metabolites between the naïve and week 1 PI mice groups. In addressing the statistical analysis across different segments of the study, varying statistical approaches were employed based on their capacity to generate the most comprehensive or significant list of metabolites, effectively increasing statistical power. Given the small sample size, traditional p-value reliance can be restrictive; therefore, we opted for “effect size” measures such as Cohen’s d and fold change to emphasize the practical significance of our findings (20). This approach underscores the relevance of effect size over conventional significance in cases of limited sample numbers. Full statistical details are provided in **Supplementary Tables 1**, **2**. It is also important to note that no p-values remained significant after adjusting for multiple testing, highlighting the importance of considering effect sizes in our analysis.

For all immunological data, statistical analysis was conducted using GraphPad Prism 8 software (version 8.4.2). Data are presented as mean ± standard error of the mean (SEM). The unpaired Student’s t-test with unequal variance (two-tailed) determined statistical significance between groups. Statistical significance was denoted as * for p ≤ 0.05, ** for p ≤ 0.01, and *** for p ≤ 0.001.

## 3 Results

### 3.1 SSG-NIV treatment resulted in significantly lower *L. donovani* parasite burdens in infected mice

**Figure 1:**
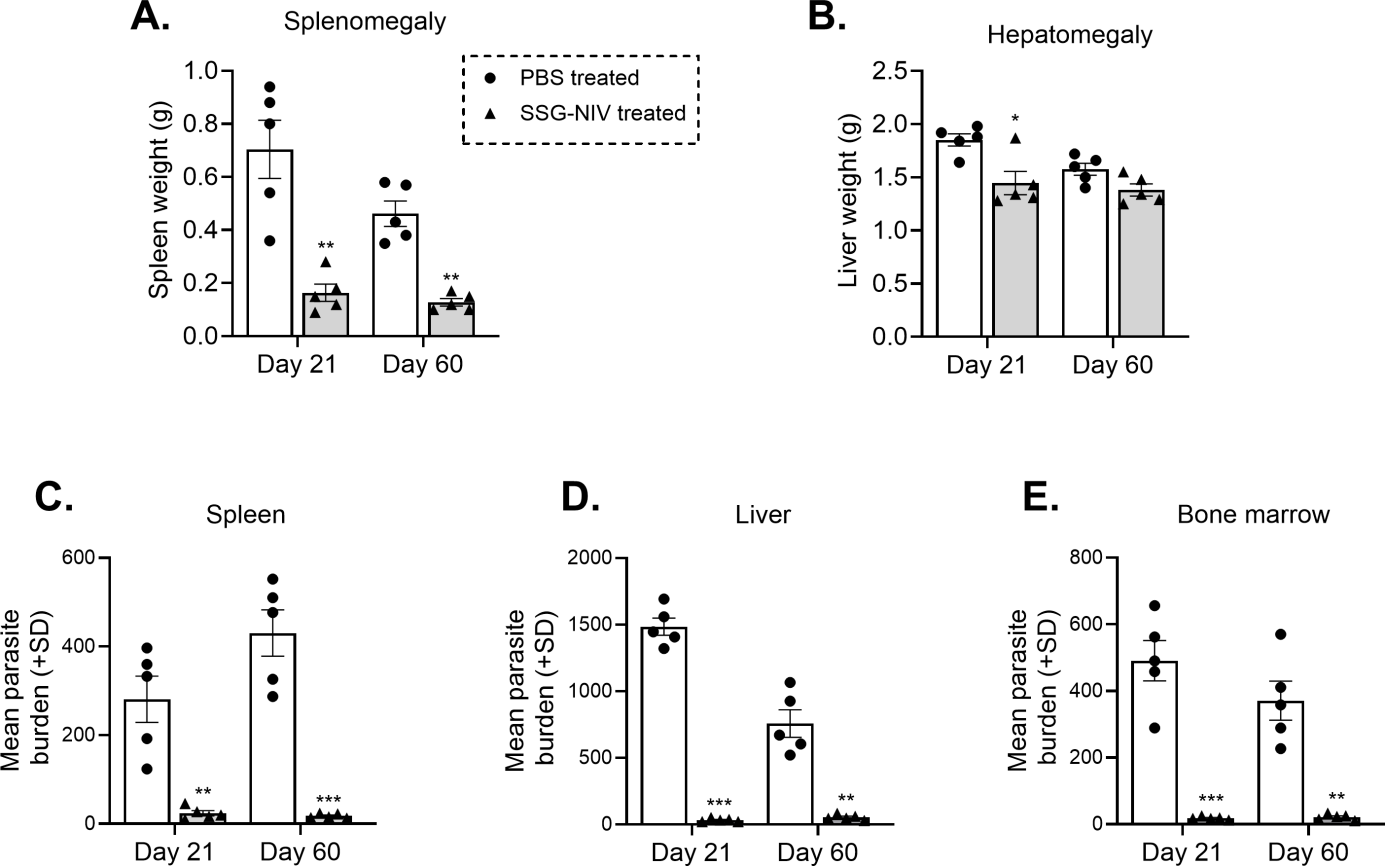
Treatment of *L. donovani* infected BALB/c mice with SSG-NIV significantly decreased parasite burden in tissues. BALB/c mice were infected with 2 × 10^7^ *L. donovani* amastigotes and subsequently treated with either SSG-NIV (300 mg Sb^v^/kg) or given PBS (as controls). Animals were sacrificed on days 21 and 60 post-infection (PI), and the effects of the drug was assessed on **(A)** spleen weight, and **(B)** liver weight. Parasite burdens were determined by LDU in the **(C)** spleen, **(D)** liver, and **(E)** bone marrow. Data represents findings from two independent experiments (n = 5 per group). Statistical analysis was conducted using Student’s t-test, comparing differences to the relevant control mice at days 21 and 60 (*p ≤ 0.05; **p ≤ 0.01; ***p ≤ 0.001).

BALB/c mice infected with *L. donovani* and treated with 300 mg Sb^v^/kg of SSG-NIV were compared to controls at day 21 and day 60 PI. At day 21 PI, mice treated with SSG-NIV exhibited reductions in spleen (**Figure 1A**) and liver (**Figure 1B**) weights. At the late stage of infection, the SSG-NIV-treated mice consistently maintained significantly smaller spleen weights, but their hepatic weight at day 60 was comparable to that of the control mice. The LDU was used to assess parasite burdens in the spleen, liver, and bone marrow at days 21 and 60 PI. As previously reported by (10), mice infected with *L. donovani* and treated with SSG-NIV exhibited a significant reduction in parasite burden at day 21 in the spleen (**Figure 1C**), liver (**Figure 1D**), and bone marrow (**Figure 1E**). This reduction in parasite burden was maintained up to day 60 PI. The control mice showed a lower number of hepatic parasites at day 60 compared to day 21 (**Figure 1D**).

### 3.2 Splenocytes from *L. donovani* SSG-NIV treated mice produced significantly lower amounts of IL-10 and TGF-B compared with cells from infected control mice

**Figure 2:**
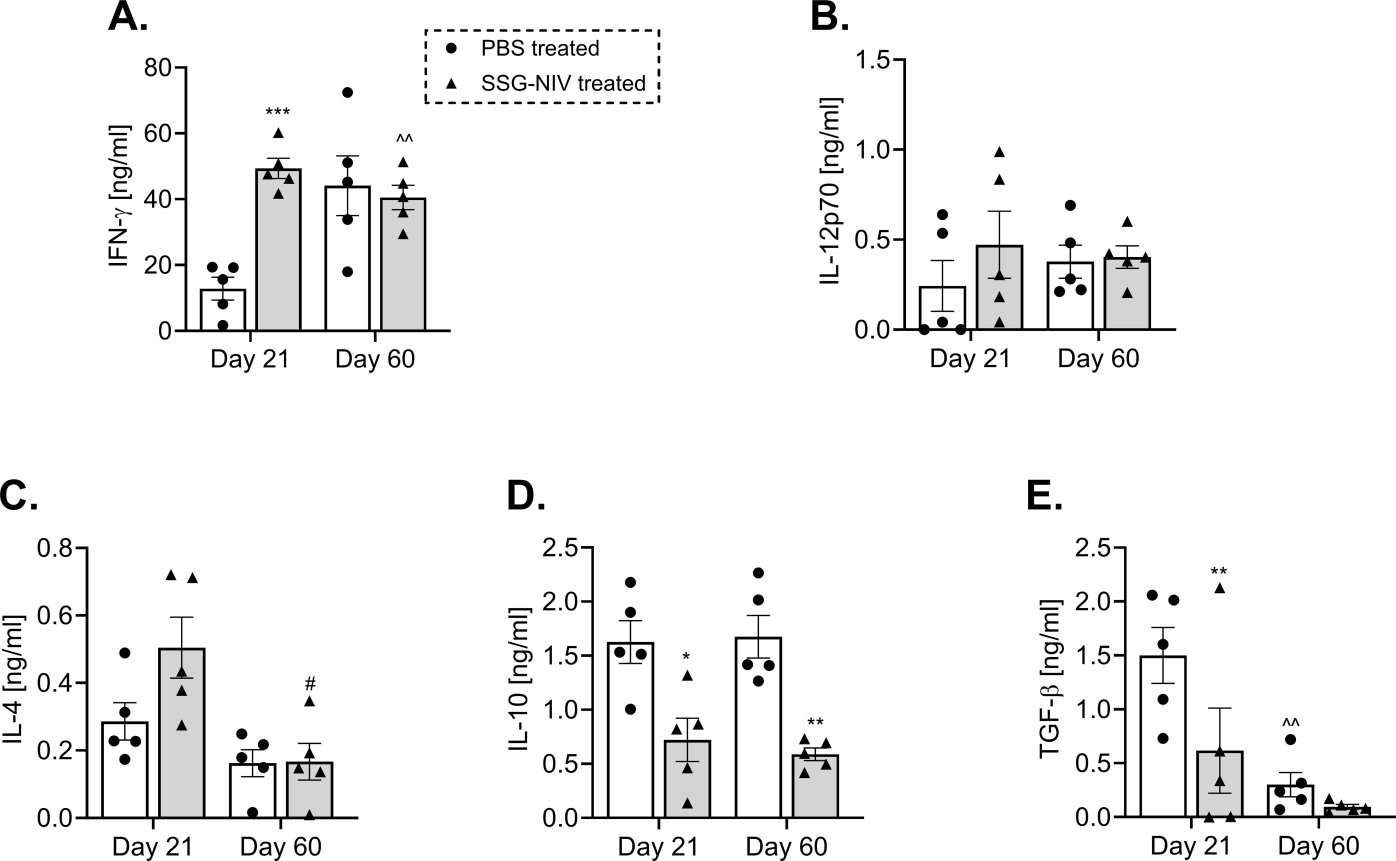
*L. donovani* infected BALB/c mice treated with SSG-NIV exhibited heightened type 1 immune response and diminished type 2 immune response. BALB/c mice infected with 2 × 10^7^ *L. donovani* amastigotes were treated with either SSG-NIV (300 mg Sb^V^/kg) or PBS on day 7 PI. At days 21 and 60 PI, spleen cells were stimulated with 20 µg/ml α-CD3 for 72 hours. The production of **(A)** IFN-γ, **(B)** IL-12p70, **(C)** IL-4, **(D)** IL-10, and **(E)** TGF-β was measured in cell supernatants by ELISA. Results are representative of two independent experiments (n = 5 per group), and data were analyzed using Student’s t-test. Statistical significance was determined by comparing differences to control mice (*p ≤ 0.05, **p ≤ 0.01, ***p ≤ 0.001), with #p ≤ 0.05 for day 21 SSG-NIV compared to day 60 SSG-NIV-treated mice, and ^^p ≤ 0.01 for day 21 control compared with day 60 control.

During VL, lymphocytes’ predominant production of various cytokines such as IFN-γ, IL-12, and IL-4 (21) has been associated with protection, whereas predominant production of IL-10 and TGF-β are susceptibility factors (5, 21). In this study, administration of SSG-NIV significantly increased the levels of protective cytokines. At day 21, the concentration of IFN-γ was significantly higher in the SSG-NIV-treated mice when compared to the control group, but no significant differences were observed at day 60 (p < 0.001; **Figure 2A**). Although not statistically significant, SSG-NIV treatment also showed a trend for elevated levels of IL-12p70 and IL-4, two other protective cytokines in VL (**Figures 2B, C**). We observed comparable levels of protective cytokines at day 60 PI between the two groups of mice, showing a drop in the liver parasite burden in both groups. In contrast, the SSG-NIV-treated mice exhibited significantly reduced levels of immunosuppressive cytokines, IL-10 and TGF-β, at all-time points (**Figures 2D, E**).

### 3.3 SSG-NIV-treated *L*. *donovani* mice show higher spleen CD4^+^ and CD8^+^ IFN-γ-producing cells than infected controls

**Figure 3:**
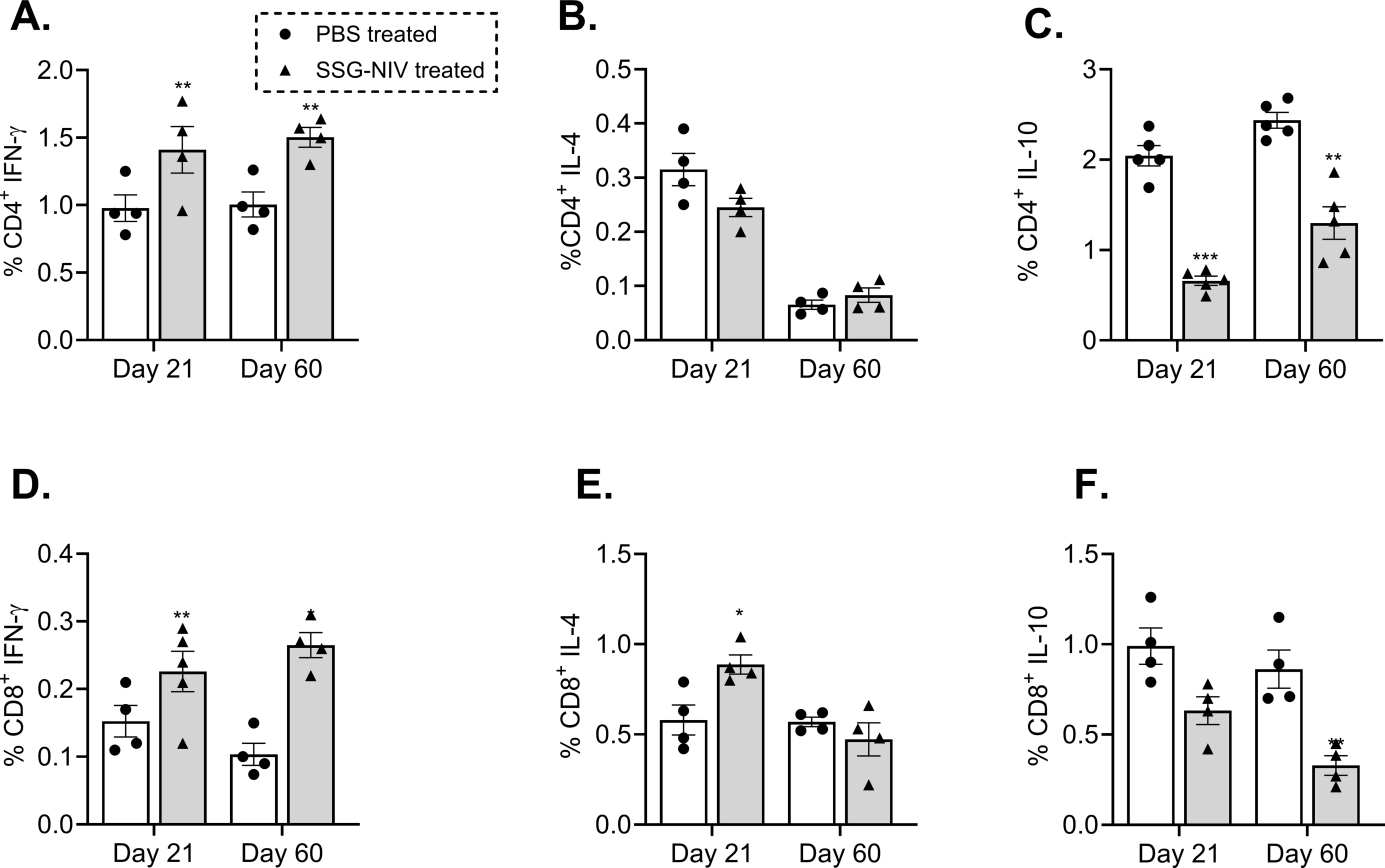
Frequency of cytokine producing T cells from splenocytes of mice infected with *L. donovani.* BALB/c mice were infected with 2 × 10^7^ *L. donovani* amastigotes and treated on day 7 with either SSG-NIV (300 mg SbV/kg) or PBS via the lateral tail vein. At days 21 and 60 PI, frequency of **(A)** CD4^+^IFN-γ^+^, **(B)** CD4^+^IL-4^+^, **(C)** CD4^+^IL-10^+^, **(D)** CD8^+^ IFN-γ^+^, **(E)** CD8^+^IL-4^+^, and **(F)** CD8^+^IL-10^+^ was determined in spleen cells by flow cytometry. Data from two independent experiments (n = 5 per group) were analyzed using a Student’s t-test (± SEM). Statistical significance was determined compared to control group (*p ≤ 0.05; **p ≤ 0.01). ***P ≤ 0.001.

Both CD4^+^ and CD8^+^ T cell populations are indispensable for a successful therapy outcome with SSG (22, 23). Thus, we examined the percentage of CD4^+^ and CD8^+^ T cell populations producing IFN-γ, IL-10, and IL-4 within the spleens of the *L. donovani* SSG-NIV-treated and control mice. Our results showed that the SSG-NIV-treated mice exhibited significantly higher levels of IFN-γ producing CD4^+^ T cells at both days 21 and 60 PI compared to the control group (p < 0.01; **Figure 3A**). The frequency of CD4^+^IL-4^+^ cells was comparable between the two groups of mice at both time points (**Figure 3B**). Importantly, the expression of IL-10, an immunosuppressive cytokine (24), was significantly lower in the SSG-NIV-treated mice at both time points compared to the control group (**Figure 3C**). For CD8^+^ T cells, we observed significantly higher levels of IFN-γ production in the SSG-NIV-treated mice at both days 21 and 60 PI. (**Figure 3D**). Similarly, the expression of IL-4 was significantly higher in the SSG-NIV-treated mice at day 21 but not at day 60 PI (**Figure 3E**). Furthermore, the SSG-NIV-treated mice exhibited lower levels of IL-10 compared to the control group on both days 21 and 60 PI (**Figure 3F**). Geometric mean fluorescence intensity (gMFI) analysis revealed that SSG-NIV treated mice had significantly higher levels of CD4^+^ IFN-γ^+^ (p ≤ 0.05), CD4^+^IL-4^+^ (p ≤ 0.05), CD8^+^ IFN-γ^+^ (p ≤ 0.01) and CD8^+^ IL-4^+^ (p ≤ 0.05) cells compared to controls on day 21, with levels becoming comparable by day 60 (**Supplementary Figure 1**). Additionally, gMFI analysis showed that CD4^+^ T cells produced more IL-4 and IL-10 per cell, whereas CD8^+^ T cells exhibited higher per-cell production of IFN-γ, underscoring the distinct roles of these subsets in the host’s immune response against *L*. *donovani.*

### 3.4 Enhanced nitrite is associated with healing phenotype in SSG-NIV-treated mice during *L. donovani* infection

**Figure 4:**
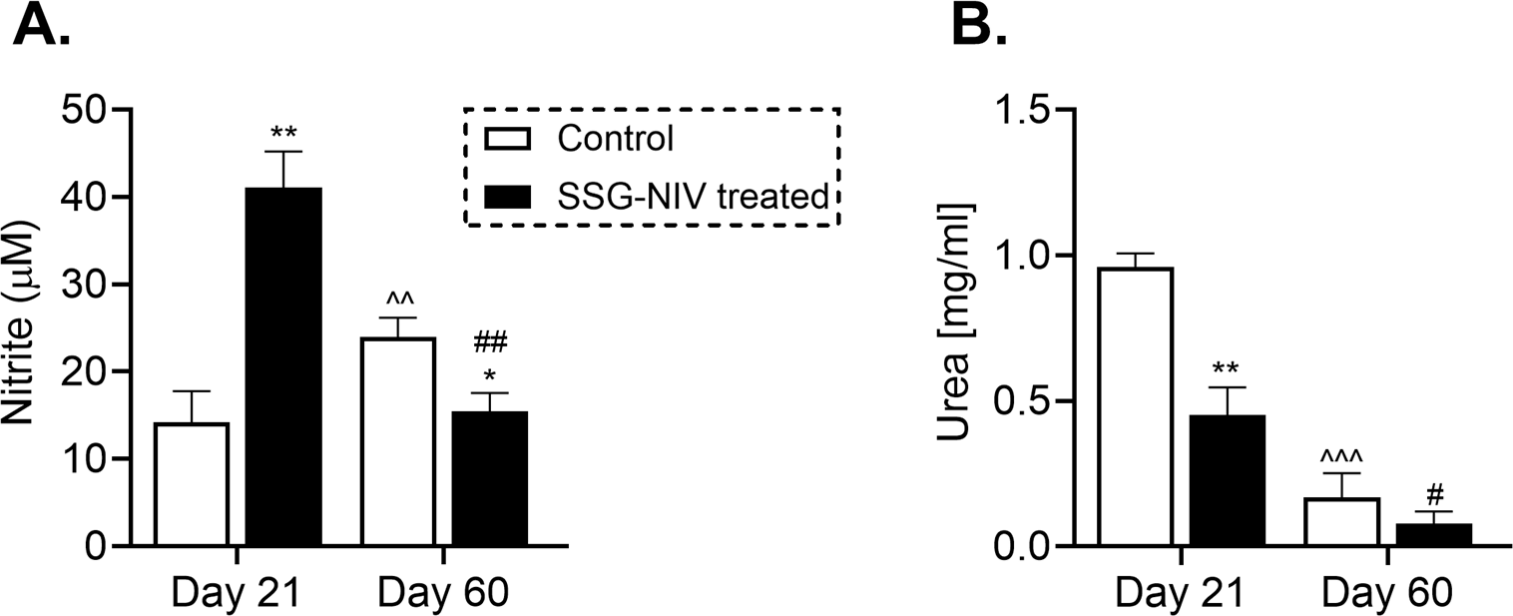
The SSG-NIV treated *L. donovani* infected BALB/c mice exhibited elevated nitrite levels and concomitant reductions in urea concentrations. BALB/c mice were infected with 2 × 10^7^ *L. donovani* amastigotes and treated on day 7 with either SSG-NIV (300 mg Sb^V^/kg) or PBS via the tail vein. The production of nitrite and urea was assessed in splenocytes at days 21 and 60 PI. **(A)** Nitrite levels were measured using a Griess assay, and **(B)** arginase activity assay measured urea production. Data represent findings from two independent experiments (n = 5 per group). Statistical significance was determined by comparing the differences in the SSG-NIV treated mice to control mice at days 21 and 60 PI (*p ≤ 0.05, **p ≤ 0.01), with #p ≤ 0.05 for day 21 SSG-NIV compared with day 60 SSG-NIV-treated, ##p ≤ 0.01 indicating significant changes from day 21 to day 60 PI for the SSG-NIV-treated group, and ^p ≤ 0.05; ^^p ≤ 0.01 for day 21 control compared with day 60 control.

To elucidate the underlying mechanisms contributing to reduced parasite burdens in SSG-NIV-treated mice, we measured nitrite and urea levels. Nitric oxide, a crucial molecule for *Leishmania* killing (5), was significantly higher in spleen cell supernatants of SSG-NIV-treated mice at day 21 PI, but decreased by day 60 PI compared to PBS-treated animals (**Figure 4A**). In contrast, the control group displayed a slight increase in nitrite levels from day 21 to day 60 PI (**Figure 4A**). We also observed a significant reduction in arginase activity in splenocytes from SSG-NIV-treated mice at day 21 PI, while no differences were detected at day 60 PI (**Figure 4B**).

### 3.5 SSG-NIV treatment alters the recruitment and activation of macrophages and dendritic cells in *L. donovani*-infected mice

**Figure 5:**
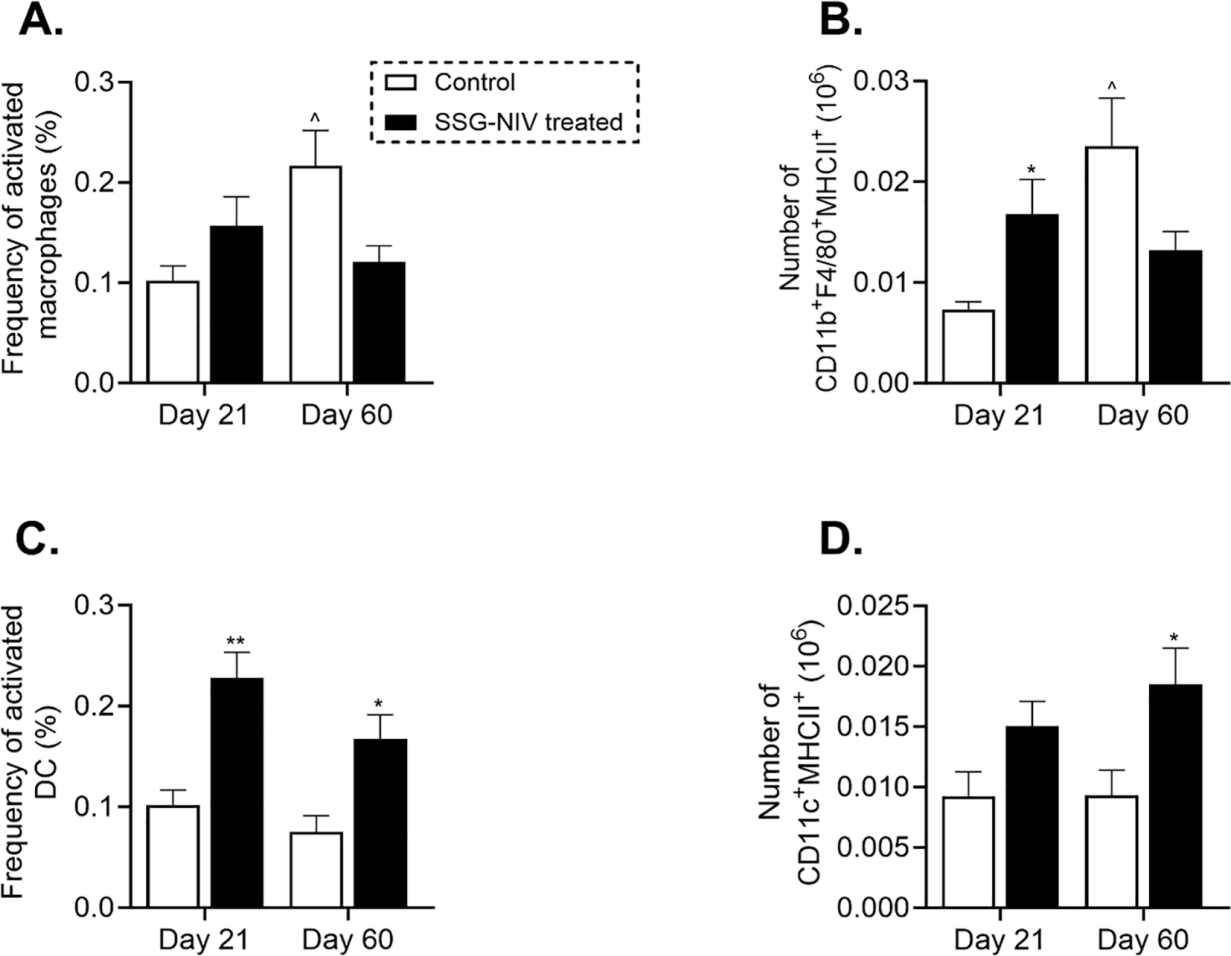
Myeloid cell populations in *L. donovani*-infected mice. BALB/c mice were infected with 2 × 10^7^ *L. donovani* amastigotes and treated on day 7 with either SSG-NIV (300 mg Sb^V^/kg) or PBS via the tail vein. The frequency and absolute number of CD11b^+^F4/80^+^MHCII^+^ macrophages (**A, B**, respectively), as well as frequency and absolute number of CD11c^+^MHCII^+^F4/80^-^ DCs (**C, D**, respectively) were determined in splenocytes. Data from two independent experiments (n = 5 per group) were analyzed using a Student’s t-test. Statistical significance was determined compared to control mice at days 21 and 60 PI (*p ≤ 0.05; ** p ≤ 0.01).

Myeloid cells play crucial roles in the persistence and clearance of *Leishmania* within the host (5). In this study, we assessed the recruitment and activation of myeloid cells during infection and drug treatment. We found that infiltration of activated macrophages (CD11b^+^F4/80^+^MHCII^+^) was higher in the SSG-NIV-treated group compared to control mice, although the difference was not statistically significant at day 21 PI (**Figure 5A**). However, macrophage levels declined by day 60 PI compared to day 21 PI in the SSG-NIV-treated group (**Figure 5A**). Notably, the absolute cell counts of activated macrophages in the SSG-NIV-treated mice was significantly higher at day 21 PI but decreased by day 60 PI compared to the control group (**Figure 5B**). Treatment with SSG-NIV led to a significant increase in the percentage of activated DCs (CD11c^+^MHCII^+^F4/80^-^) infiltrating the spleen at day 21 PI, a trend that persisted until day 60 PI. (**Figure 5C**). Similarly, the absolute cell counts of activated DCs were increased in SSG-NIV-treated mice at both days 21 and 60 PI. (**Figure 5D**). Overall, SSG-NIV treatment in BALB/c promoted *in vivo* trafficking and expansion of activated macrophages and DCs.

### 3.6 SSG-NIV administration favors early recruitment and activation of CD4^+^ T cells in *L. donovani-*infected mice

**Figure 6:**
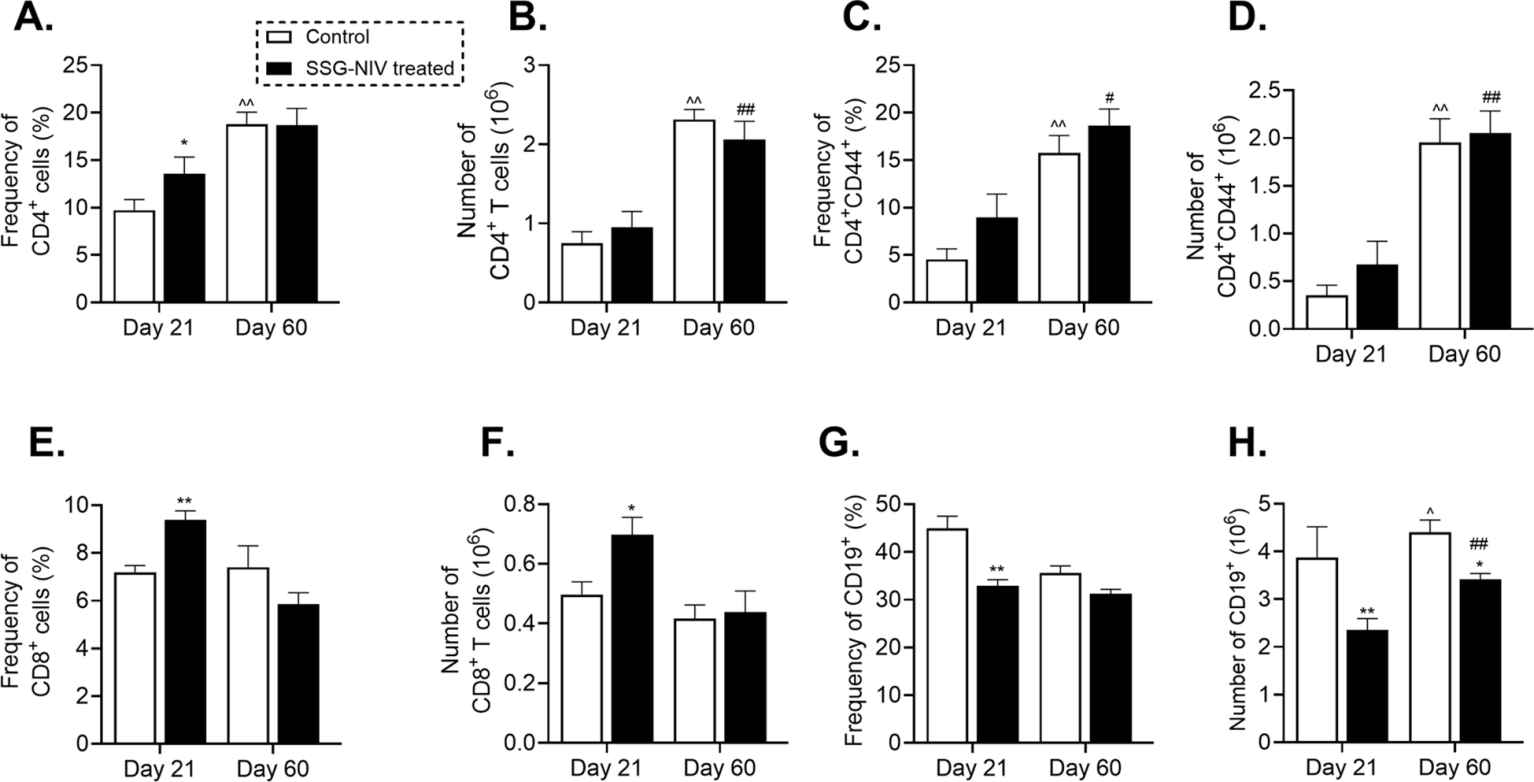
Frequency of lymphocytes population in *L. donovani*-infected mice. BALB/c mice were infected with 2 × 10^7^ *L. donovani* amastigotes and treated on day 7 with either SSG-NIV (300 mg SbV/kg) or PBS via the lateral tail vein. At days 21 and 60 PI, the **(A)** frequency CD3^+^CD4^+^, **(B)** absolute CD3^+^CD4^+^, **(C)** frequency CD4^+^CD44^+^, **(D)** absolute CD4^+^CD44^+^, **(E)** frequency CD3^+^CD8^+^, **(F)** absolute CD3^+^CD8^+^, **(G)** frequency CD3^-^CD19^+^, and **(H)** absolute CD3^-^CD19^+^ were determined in spleen cells by flow cytometry. Data from two independent experiments (n = 5 per group) were analyzed using a Student’s t-test. Statistical significance was determined by comparing differences to control mice (*p ≤ 0.05; **p ≤ 0.01), with #p ≤ 0.05 for day 21 SSG-NIV compared with day 60 SSG-NIV-treated, ##p ≤ 0.01 indicating significant changes from day 21 to day 60 PI for the SSG-NIV-treated group and ^p ≤ 0.05; ^^p ≤ 0.01 for day 21 control compared with day 60 control.

To further understand the immune response associated with SSG-NIV treatment in BALB/c mice, we examined spleen immune cell recruitment using flow cytometry, detailed in **Supplementary Figure 2**. At day 21 PI, SSG-NIV-treated mice showed higher percentages of CD3^+^CD4^+^ T helper (Th) cells and activated T cells (CD4^+^CD44^+^) compared to the control group, with only the CD3^+^CD4^+^ subset showing significant differences (**Figure 6A, C**). By day 60 PI, these percentages were similar between the two groups. Effector Th cells significantly increased by day 60 PI in both SSG-NIV and control groups, indicating more T cell activation in later infection stages, with or without treatment (**Figure 6C**). When considering absolute cell counts of Th and effector T cells, both treated and control mice had comparable numbers at both time points, but within each group, levels significantly increased at day 60 compared to day 21 PI (p ≤ 0.01; **Figures 6B, D**). CD8^+^ T cells are also implicated in control of VL (25). The percentages and absolute cell numbers of CD8^+^ T cells were significantly elevated in the SSG-NIV-treated group on day 21 but not on day 60 PI (p ≤ 0.01 in **Figure 6E** and p ≤ 0.05 in **6F**). At day 21 PI, the SSG-NIV-treated mice exhibited a significant decrease in the percentage and cell count of B cells compared to the control group (p ≤ 0.01; **Figures 6G, H**). However, the absolute number of B cells increased significantly from day 21 to day 60 PI in both the SSG-NIV-treated and control groups.

### 3.7 Identification of potential markers of early infection in experimental visceral leishmaniasis

**Figure 7:**
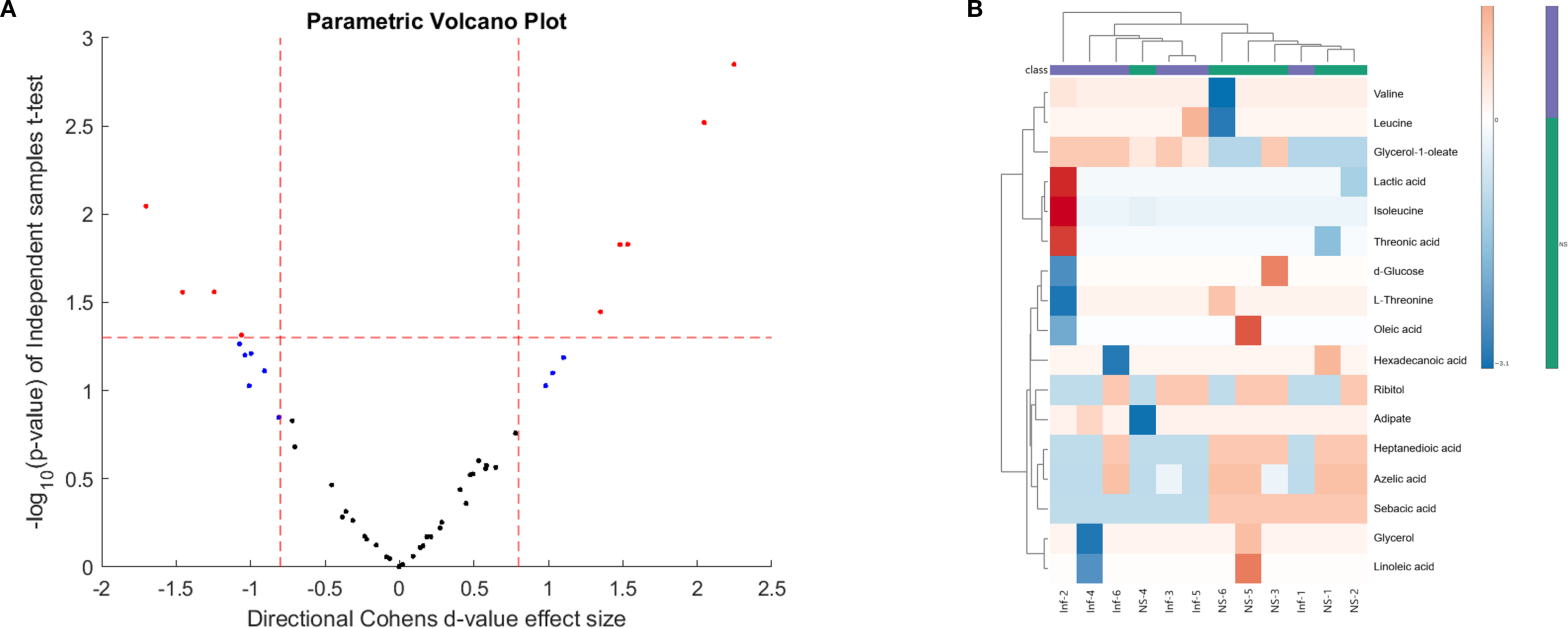
**(A)** Volcano plot and **(B)** heatmap comparing the sera of naïve (NS) and week 1 PI (Inf) mice. BALB/c mice were infected with 2 × 10^7^ *L. donovani* amastigotes and sacrificed at week 1 PI. GCxGC TOFMS was performed of sera in 8 animals per group. The volcano plot displays -log_10_ (p-value) against Cohen’s d-values, highlighting statistically significant metabolites (red dots) differentiating the two groups (n = 8 per group).

Immune cell activation, proliferation, and fate, are tightly related to the activation of specific intrinsic metabolic processes (26). These alterations in the immune cell metabolome can dictate the resulting phenotype as the end-product of gene transcription and translation. With this in mind, we sought to investigate the changes to the host metabolome in response to *L. donovani* infection and treatment in BALB/c mice. Initially, we aimed to determine if early infection (week 1 PI) with *L. donovani* was associated with changes in the BALB/c metabolome compared to naïve mice. Cohen’s d-values identified a list of metabolites (**Figure 7A**, **Supplementary Table 3**), with 17 being significantly different when comparing the two groups. *L. donovani* infection upregulated nine metabolites and downregulated eight. The data was used to produce a heat map visualization (**Figure 7B**), which highlights distinct metabolic profiles between naïve and infected mice, particularly in carbohydrates, fatty acids, and amino acids, emphasizing the metabolic shifts associated with early infection (**Figure 7B**).

### 3.8 Identification of potential markers differentiating successful treatment with SSG-NIV in experimental visceral leishmaniasis

**Figure 8:**
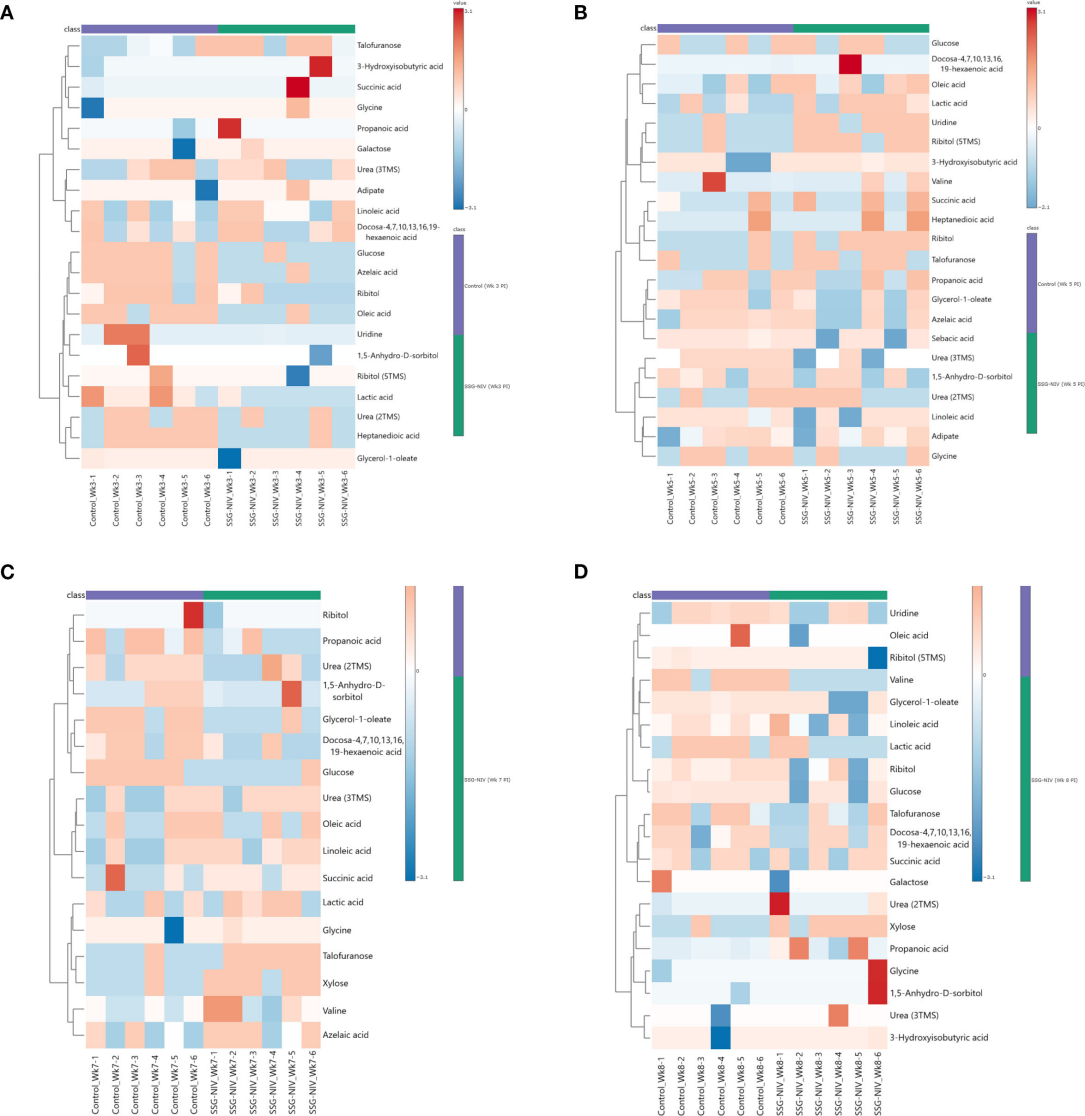
Differential metabolite markers between SSG-NIV-treated and *L*. *donovani*-infected untreated control mice. Data was visualized as heatmaps generated using MetaboAnalyst from serum samples of BALB/c mice treated with either SSG-NIV or PBS at weeks **(A)** 2, **(B)** 4, **(C)** 6, and **(D)** 7.

To identify those metabolite markers that could hold value for monitoring early and active disease and successful treatment outcomes, we analyzed metabolite changes in the SSG-NIV-treated and control (infected but untreated) mice using GC×GC-TOFMS. We used fold change (FC) to identify differential metabolites (**Figures 8A–D**) as previously described for murine studies (27–29). Metabolite analysis revealed dynamic alterations in metabolite levels at different weeks PT and PI, and are listed and visualized in **Supplementary Table 4** and **Figures 8A–D**. Using an FC ≥ 1.5 (**Supplementary Figure 3**), 24 metabolites were identified that best describe the variation between a successful treatment outcome and active disease in BALB/c mice. At week 2 PT, 6 metabolites were significantly downregulated and 18 upregulated in the SSG-NIV-treated mice compared to *L. donovani* infected and untreated animals; however, by week 4, 16 metabolites were downregulated and 8 upregulated. Additionally, talofuranose, xylose, glycine and carboxylic acids, namely, heptanedioic acid, lactic acid, propanoic acid, and sebacic acid exhibited consistent changes at weeks 2 and 4 PT. Sorbitol (decrease), galactose (increase), and glycerol-1-oleate (decrease) displayed distinct alterations at week 2 PT, and further consistent changes from weeks 4 to 8.

### 3.9 Metabolite trends as indicators of early disease and treatment success in experimental visceral leishmaniasis

For a metabolite to be considered a sensitive and specific diagnostic predictor of infection and treatment outcome, it must exhibit a consistent trend throughout the analysis, therefore we next focused on metabolites exhibiting consistent changes during early infection and throughout treatment relative to control animals.

In early infection (naïve vs. week 1PI analysis), levels of valine, lactic acid, and glycerol-1-oleate were note only elevated but also remained consistently high throughout the active disease phase, from week 1PI to week 7PT. These metabolites could therefore serve as potential diagnostic markers of early infection and could be useful for detecting and monitoring VL progression. During treatment, metabolites that exhibited a stable trend were glycine, which was consistently upregulated, and ribitol and linoleic acid, which showed a constant decrease relative to successful treatment. However, since linoleic acid was also detected in SSG-NIV treated groups, glycine and ribitol were identified as potential candidates for predicting and monitoring successful treatment during L. donovani-induced VL.

We subsequently examined metabolite pathways between SSG-NIV-treated mice and control mice. This analysis revealed significant enrichments in various pathways (**Supplementary Figure 4**), the most notable being related to the biosynthesis of neomycin, kanamycin, and gentamicin, followed by galactose metabolism and the biosynthesis of unsaturated fatty acids. Additional pathways, such as purine metabolism, aminoacyl-tRNA biosynthesis, and primary bile acid biosynthesis, were also identified as enriched. Among these, three pathways were related to fatty acid biosynthesis and metabolism, seven to carbohydrate metabolism, and five to amino acids.

## 4 Discussion

Visceral leishmaniasis, caused by *L. donovani* or *L. infantum*, is a fatal infection, with current drugs limited by complications and life-threatening side effects. Pentavalent antimonial drugs were the first line of treatment, succeeded by amphotericin B, paromomycin, and miltefosine, but the success of all regimens is hindered by toxicity, high cost, drug failure, and parasite resistance (3). Given these challenges, Baillie et al. (1985), developed a non-ionic surfactant formulation as a drug carrier system for leishmaniasis treatment. In experiments with BALB/c mice, when compared with free-SSG and AmBisome, a single dose of SSG loaded into NIV demonstrated high efficiency in treating acute leishmaniasis, leading to immune responses characteristic of a healed phenotype (12). This highlights the potential of SSG-NIV as a promising treatment option for leishmaniasis. Our aim was to identify metabolites as potential markers for active disease and treatment outcomes in VL by investigating host immune responses and metabolomes after SSG-NIV treatment.

Previous research indicates that successful drug therapy requires the involvement of CD4^+^ and CD8^+^ T cells (23, 30), particularly through the activity of IL-2, IL-4, TNF-α, IFN-γ, and IL-12, along with reactive oxygen species (ROS) and nitric oxide (NO) production (3, 5, 31). At day 21 PI, SSG-NIV treatment increased IFN-γ, IL-12, and IL-4 levels. However, by day 60 PI, cytokine levels equalized, possibly due to a balance of cytokine production by splenic CD4^+^ T cells. Indeed, IFN-γ^+^ IL-10^+^ cells have been shown to represent approximately 5% of the total CD4^+^ cell population in the spleen (32). Previous studies have shown initial high levels of these cytokines in VL patients, which significantly declined after treatment (33, 34). Despite higher levels of IFN-γ in control mice at later stages, high parasite burdens persisted due to elevated levels of IL-10, which likely dampened IFN-γ efficacy and sustained parasites in the spleen. CD8^+^ T cells contribute to IL-10 secretion (35), with IL-10 serving as a major immunosuppressive cytokine during VL by inhibiting IFN-γ production (32). Lower IL-10 levels in SSG-NIV mice may have significantly contributed to a healing phenotype, as observed in other studies (36, 37). Similarly, TGF-β, which suppresses macrophage activation and T cell proliferation thus aiding parasite proliferation (38, 39), was lower in the SSG-NIV-treated mice. The higher levels of IL-10 and TGF-β in the control group indicate their immunosuppressive roles in disease progression, while SSG-NIV treatment appears to decrease these regulatory cytokines and increase proinflammatory cytokines compared to the control group.

*Leishmania* infection induces distinct macrophage phenotypes: M1 macrophages are activated by type 1 immune responses and exhibit a proinflammatory phenotype, killing *Leishmania* through Th1 cytokines, NO, and ROS. In contrast, M2 macrophages, induced by type 2 immune responses, support parasite survival by producing Th2 cytokines and urea (40). Nanocarriers like liposomes, silver, and gold nanoparticles have demonstrated the ability to induce macrophage polarization *in vivo*, influencing cytokine secretion and cellular uptake (41). SSG-NIV treatment resulted in an increase in the absolute number of splenic macrophages, with a shift towards M1 activation and a concurrent reduction of M2 activation at day 21, as indicated by changes in nitrite and urea production, respectively. This supports (42), where nanocarriers modulated the phenotype of tumor-associated macrophages from M2 to M1. The reduced IL-10 and TGF-β levels together with increased nitrite in SSG-NIV-treated mice support the shift away from the M2 phenotype, which is promising for clinical applications against VL.

The SSG-NIV-treated mice showed elevated levels of CD4^+^ and CD4^+^CD44^+^ T cells at day 21, indicating an early activation of CD4^+^ T cells during *L. donovani* infection. CD4^+^ T cells are essential in controlling *Leishmania* infection by secreting proinflammatory cytokines like IL-12, IFN-γ, and TNF-α, which activate macrophages to produce NO and eliminate the parasites (43). This increase in CD4^+^ T cells within the SSG-NIV-treated mice supports the significant reduction in parasite burden. Similarly, CD8^+^ T cells have a crucial role in controlling experimental VL through their cytotoxic activity and IFN-γ production (44, 45). On day 21, SSG-NIV-treated mice exhibited significantly higher CD8^+^ T cell levels than the control mice. CD8^+^ T cells are known to contribute to protective immunity post-vaccination against *L. donovani*-induced VL (46), suggesting that the enhanced presence and functionality of CD8^+^ T cells in SSG-NIV-treated mice may underlie the observed reduction in parasite numbers. The observed reduction in B cells percentages in the SSG-NIV-treated mice at days 21 and 60 PI aligns with previous findings suggesting the involvement of B cells in the pathogenesis of VL (47). Studies have shown that B cell knockout mice are more protected from *L. donovani* infection compared to wild-type mice (47). Furthermore, a recent study has indicated that B cells are associated with the exacerbation of VL by inducing IL-10 (48). Therefore, the reduced presence of B cells in the SSG-NIV-treated mice supports the targeting of B cells to mitigate VL pathogenesis.

**Figure 9:**
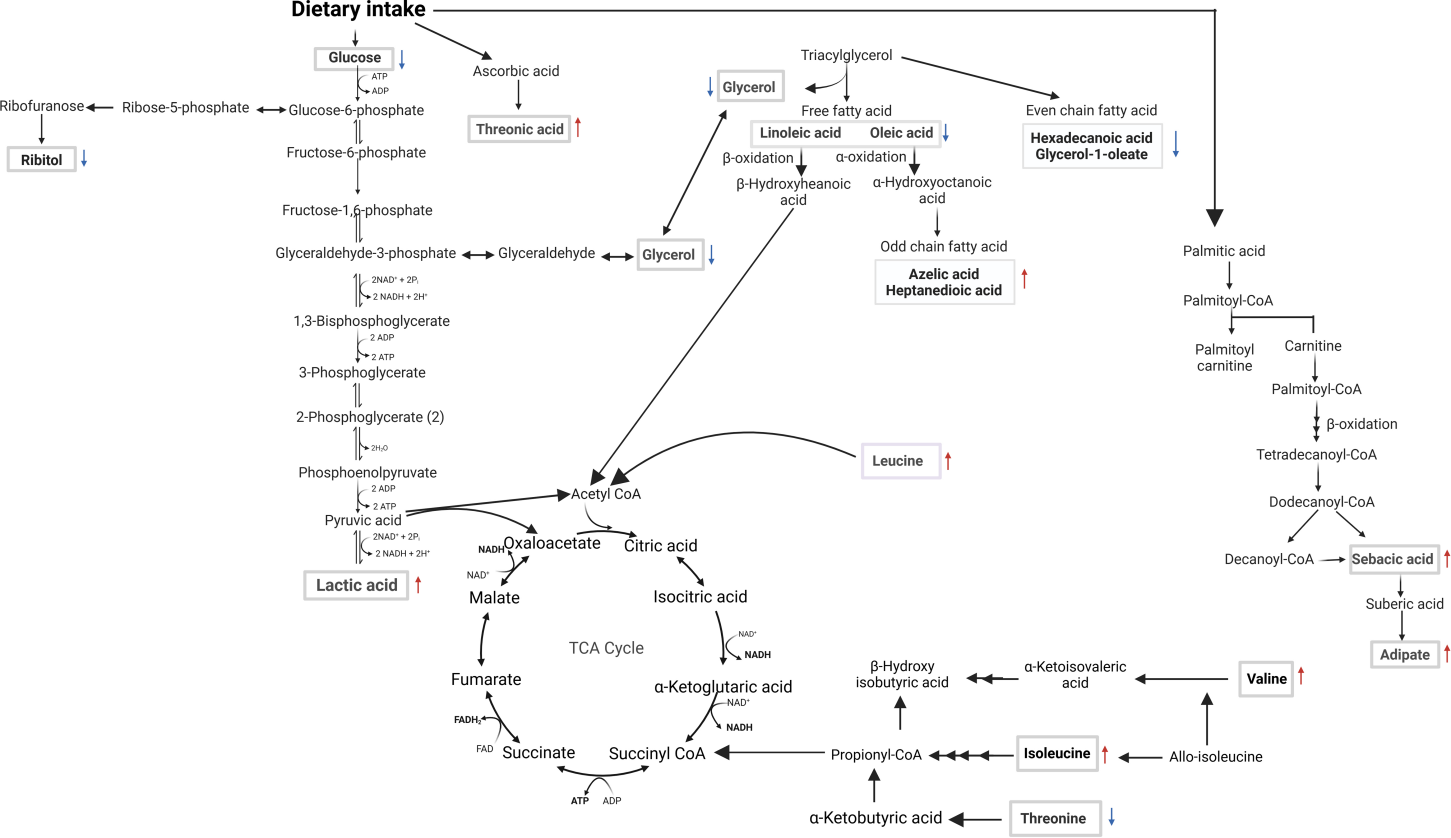
Overview of metabolomic pathway alterations in naïve vs. week 1 PI BALB/c mice. Serum samples were analyzed using GC×GC-TOFMS and MetaboAnalyst. Altered metabolites are highlighted in rectangular boxes. ATP (adenosine triphosphate), ADP (adenosine diphosphate), FAD (flavin adenine dinucleotide), FADH2 (reduced flavin adenine dinucleotide), NAD+ (nicotinamide adenine dinucleotide), NADH (reduced nicotinamide adenine dinucleotide), NADP+ (nicotinamide adenine dinucleotide phosphate), NADPH (reduced nicotinamide adenine dinucleotide phosphate), Pi (inorganic phosphate), ↑ or ↓ denotes an increase or decrease, respectively, when comparing the concentration in the week 1 PI samples to naïve samples.

**Figure 10:**
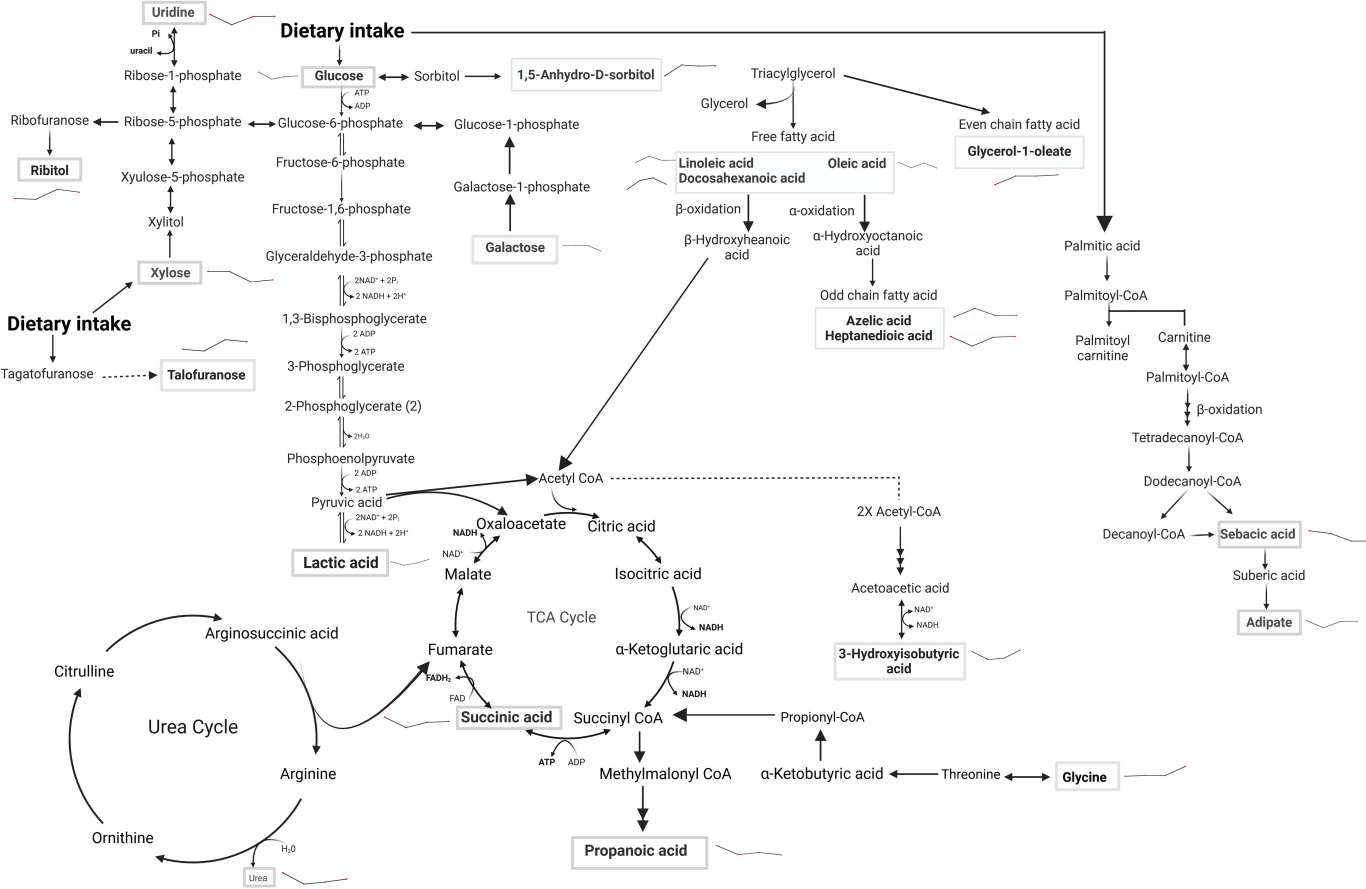
Metabolic pathway alterations in SSG-NIV vs. PBS-treated mice post-treatment. Serum samples of BALB/c mice treated with either SSG-NIV or PBS at weeks 2, 4, 6, and 7. Analysis was conducted using GC×GC-TOFMS and MetaboAnalyst. Metabolites significantly altered between the treatment groups are highlighted within rectangular boxes, showcasing the major metabolic pathways influenced by the treatment. Lines denote direction of treated group compared to the control.

Given that VL is immune-driven, changes in the metabolome could significantly impact the resulting phenotype. The parasite’s manipulation of host cell metabolism aids its survival, suggesting that targeting host metabolomics offers a potential avenue for leishmaniasis management by exploiting the competition of nutrients between host and parasite. We employed GCxGC TOFMS to identify changes in host metabolites early after infection and as a consequence of successful treatment with SSG-NIV. In an attempt to identify biological pathways in which metabolites are related, the differentially regulated metabolites were mapped onto metabolic pathways (**Figures 9**, **10**). Early after infection with *L. donovani*, metabolites covered primary metabolic pathways such as glycolysis, fatty acid metabolism and amino acid metabolism (**Figure 9**). This could be a result of increased energy demand in the host due to ongoing immune responses and *L. donovani* manipulation of host metabolic pathways to secure energy for survival and proliferation. Reduced glucose, glycerol and ribitol (**Figure 9**, **Supplementary Table 3**) support the operational Warburg effect, where increased glucose consumption by the parasite enhances energy production and virulence thereby allowing the parasite to initiate infection (49). Elevated lactic acid early after infection (**Figure 9**, **Supplementary Table 3**) may reflect a parasite induced effect as lactate dehydrogenase has been reported in the sera of VL patients’ pre-therapy compared to healthy subjects (50).

While *Leishmania* can engage in FA biosynthesis, it can also scavenge the same from the host to fulfill lipidomic needs. It is therefore not surprising that our metabolite profiles (**Figure 9**, **Supplementary Table 3**) revealed altered levels of free fatty acids, as well as even and odd-chain fatty acids (OCFA) early after infection. The role of polyunsaturated fatty acids, derived from linoleic acid have been emphasized in macrophage function and immune responses (51). For example, administration of linoleic acid enhanced Th1 immune responses and reduced the burden of *L. donovani* in mouse macrophages. This effect was associated with a decrease in expression of IL-10 and arginase (52). Amino acids are biologically important for host-parasite interaction because they are vital for protein synthesis needed by both organisms for survival. Threonic acid, branched-chain amino acids (valine, isoleucine, and leucine), and reduced threonine were detected in mice week 1 PI (**Figure 9**, **Supplementary Table 3**). A similar pattern was noted in a previous study on global metabolites in mice infected with *Trypanosoma brucei*, suggesting a compensatory mechanism to maintain the host’s energy homeostasis (53). The increased concentration of threonic acid 1 week PI (**Figure 9**, **Supplementary Table 3**), may suggest enhanced ascorbic acid metabolism, which has been shown to serve as a potent defense against various oxidants (54). Taken together, increased threonic acid at week 1 PI could be due to the parasite inducing oxidative stress in the mice tissues.

Following treatment with SSG-NIV in *L. donovani*-infected mice, metabolites covered primary metabolic pathways such as nucleic acid metabolism, glycolysis, fatty acid metabolism, amino acid metabolism, and the urea and TCA cycle (**Figure 10**).

Changes in carbohydrate levels (glucose, lactic acid, galactose, 1,5-anhydro-D-sorbitol, talofuranose, and xylose) were consistently observed at weeks 2, 4, 6, and 7 after treatment (**Figure 10**, **Supplementary Table 4**). Glucose, being a strong energy source had elevated levels at weeks 2, 6, and 7, but decreased levels at week 4, in SSG-NIV-treated mice. The elevated levels of glucose could reflect the energy demands associated with ongoing immune responses in the host. Hence, the reduction in parasite burden in SSG-NIV mice. Modulating energy metabolism offers immune cells functional advantages by supplying precursors for fatty acid and protein synthesis, crucial for membrane restructuring and cytokine production (55). Studies have shown that *L*. *infantum* triggers a rapid macrophage metabolism shift towards the Warburg effect, coupled with reduced mitochondrial activity (56). The higher lactic acid levels in control mice at weeks 5 and 7 indicate that Warburg effect is active, with the parasite consuming more glucose to enhance energy production and increase virulence (57). As alluded to above, elevated lactic acid appears to correlate with *Leishmania* infection. It was therefore not surprising that increased lactic acid persisted early after treatment commenced (week 2) but then declined as treatment progressed (weeks 4 and 6). Interestingly, SSG-NIV treatment (**Figure 10**) revealed certain carbohydrates (galactose, 1,5-anhydro-D-sorbitol, talofuranose, and xylose) that were absent during the pre-treatment stage (week 1 PI; **Figure 10**). This metabolic dynamic could signify treatment-induced shifts in host-parasite interactions.

Aligned with our immunological findings, reduced levels of galactose in the SSG-NIV-treated mice from week 4 onwards may reflect an impact of galactose on T cell functionality (**Figure 10**, **Supplementary Table 4**). A study found that T cells cultured in the presence of galactose had a severe impairment in IFN-γ production (58) thereby suggesting that reduction in galactose may favor Th1 cells. Xylose concentrations were higher in SSG-NIV-treated mice early after treatment but declined as treatment progressed. Previous findings indicated that concentrations of xylose increased significantly in human VL patients after AmpB treatment (59).

Our investigation revealed that uridine, was increased in serum upon SSG-NIV treatment in BALB/c mice (**Figure 10**, **Supplementary Table 4**). Interestingly this nucleoside was absent in early infection (**Figure 9**), at the pre-treatment stage. SSG-NIV-treated mice showed consistently higher uridine levels PT, with the exception of week 4 PT. Physiologically, elevated uridine promotes tissue and cell regeneration *in vivo* (8). This aligns with the significant reduction in spleen and liver pathology observed in SSG-NIV-treated mice. Uridine also intensifies protective immune responses. For instance, treatment with uridine diphosphate induced proliferation of CD8^+^ T cells with type I characteristics (IFN-γ and T-bet) in peripheral mononuclear blood cells and C57BL6 mice, potentially positioning this metabolite as a crucial immune mediator and enhancer of drug efficacy (60). The significantly reduced parasite burden reported in the SSG-NIV-treated mice is further supported by elevated levels of succinic acid (**Figure 10**, **Supplementary Table 4**). Interestingly, succinic acid has been associated with DC-derived proinflammatory cytokines. Treatment with SSG-NIV significantly activated splenic infiltrating DCs on day 21. This is beneficial because DCs also promote the activation of effector T cells and the production of key molecules, such as IL-12 and iNOS, essential for parasite clearance (61, 62). Reduced concentration of oleic acid at three time points (**Figure 10**, **Supplementary Table 4**), aligns with findings from a previous study (63), suggesting a role for oleic acid in mediating anti-inflammatory responses in immune cells by elevating M2 polarization.

Various essential amino acids that were increased early after infection were not detected as significantly altered after treatment with SSG-NIV (**Figure 10**, **Supplementary Table 4**), except for elevated levels of glycine throughout all weeks after SSG-NIV therapy in BALB/c mice (**Figure 10**, **Supplementary Table 4**). Glycine is known to have anti-inflammatory and immunomodulatory properties (64) and its role in infectious diseases is also evident. For instance, a previous study observed glycine enrichment in tissue and biofluids from *L. donovani*-infected BALB/c mice following treatment with miltefosine (27). Thus, the steady increase in glycine during SSG-NIV treatment indicates its potential as a prognostic marker for successful therapeutic outcomes.

## 5 Conclusions

In conclusion, our study highlights the potential of SSG-NIV as a promising treatment for visceral leishmaniasis. It demonstrates the ability to reduce parasite burden and induce complex immunological changes in *L. donovani*-infected BALB/c mice. The elevation of proinflammatory cytokines, reduction of regulatory cytokines, and modulation of T cell responses reveal the immunomodulatory potential of SSG-NIV therapy. The identification of potential metabolites underscore an intricate interplay between host immune responses and metabolism Metabolites like glucose, lactic acid, xylose, succinic acid, ribitol, and glycine exhibit pivotal roles in this interplay, offering avenues for further investigation. Incorporating these findings into the broader landscape of VL research adds depth to exploring the efficacy SSG-NIV therapeutics. As we navigate the complex terrain of VL management, the insights gleaned from this study hold promise not only in refining current treatments but also in paving the way for future diagnostic tools and therapeutic innovations. By bridging the gaps between immunology, metabolism, and therapy, our research contributes to the collective effort to alleviate the burden of this challenging disease and with further research, potentially improve patient outcomes.

## Data Availability

All data related to this article and its **Supplementary Material** are free to obtain. Datasets are available on Biostudies, using accession number S-BSST2167.
