# Supplementary material for: The effect of treatment with a non-ionic surfactant vesicular formulation of sodium stibogluconate on host immune responses and serum metabolites in a murine model of Leishmania donovani
Source: Front Immunol. 2025 Oct 2;16:1499513. doi: 10.3389/fimmu.2025.1499513 (PMC12528065; doi:10.3389/fimmu.2025.1499513)
Supplement: Supplementary file 1 [file Table1.docx]

**Supplementary Figures:**


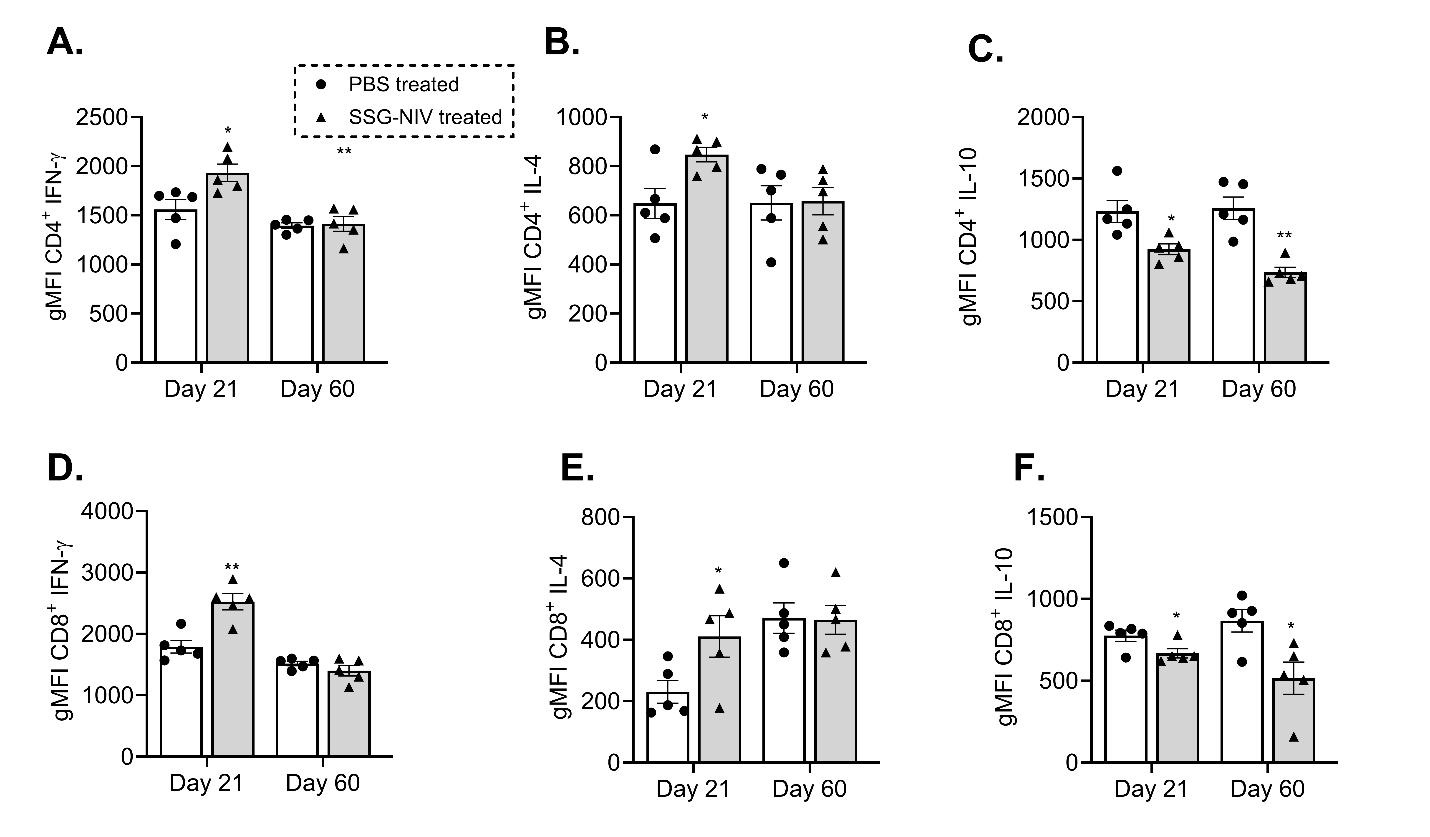


**Supplementary Figure 1:** Distinct cytokine production profiles of CD4⁺ and CD8⁺ T cells during *L*. *donovani* infection as revealed by gMFI analysis.


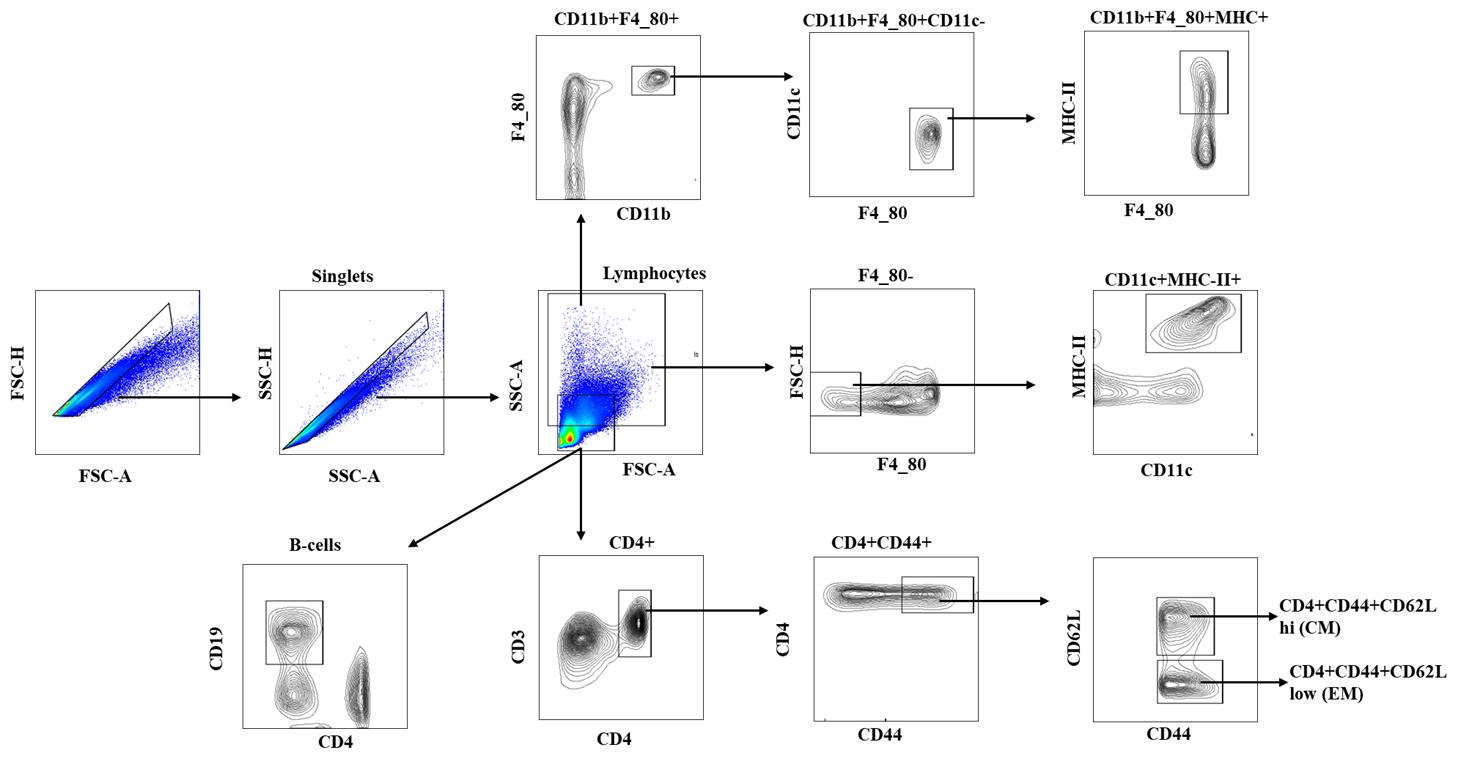


**Supplementary Figure 2**: Gating strategies used to analyse CD3^+^CD4^+^ T helper, activated T cells (CD4^+^CD44^+^), CD3^-^CD19^+^ (B cells), activated macrophages (CD11b^+^F4/80^+^MHCII^+^) and activated DCs (CD11c^+^MHCII^+^F4/80^-^).


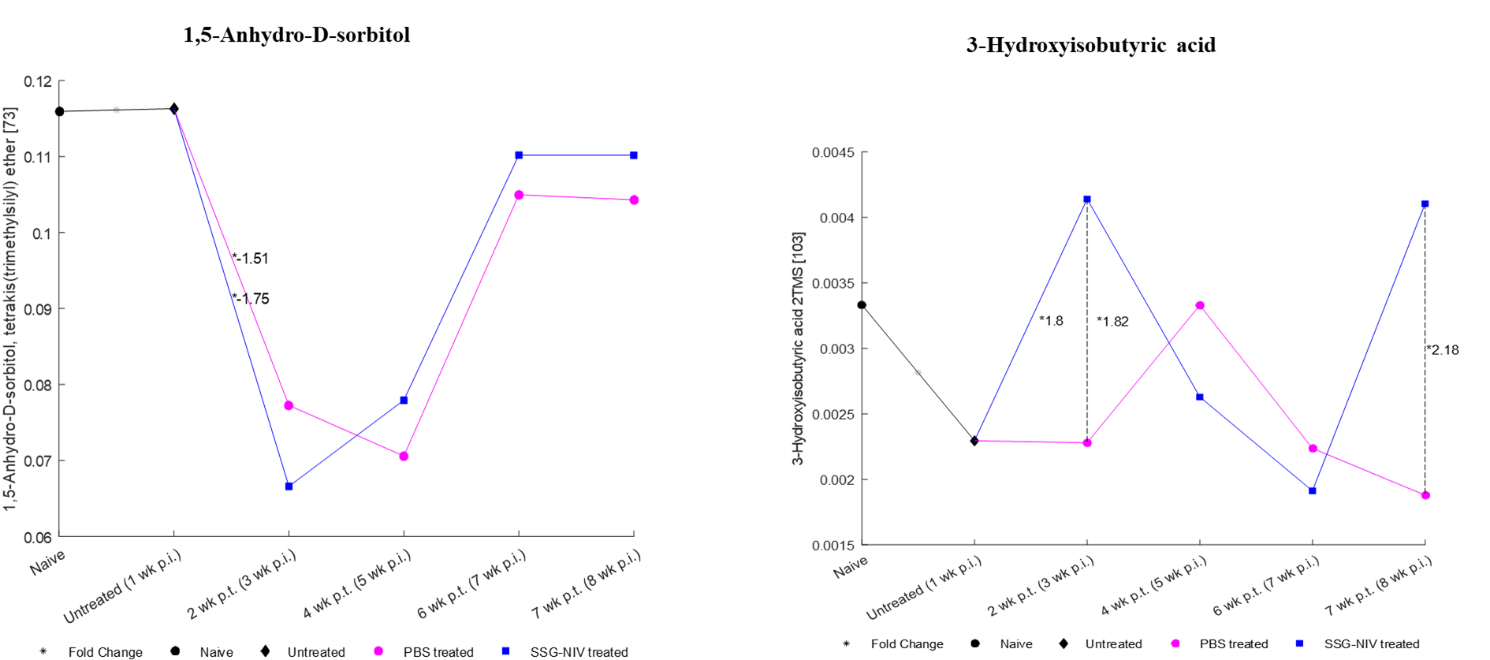


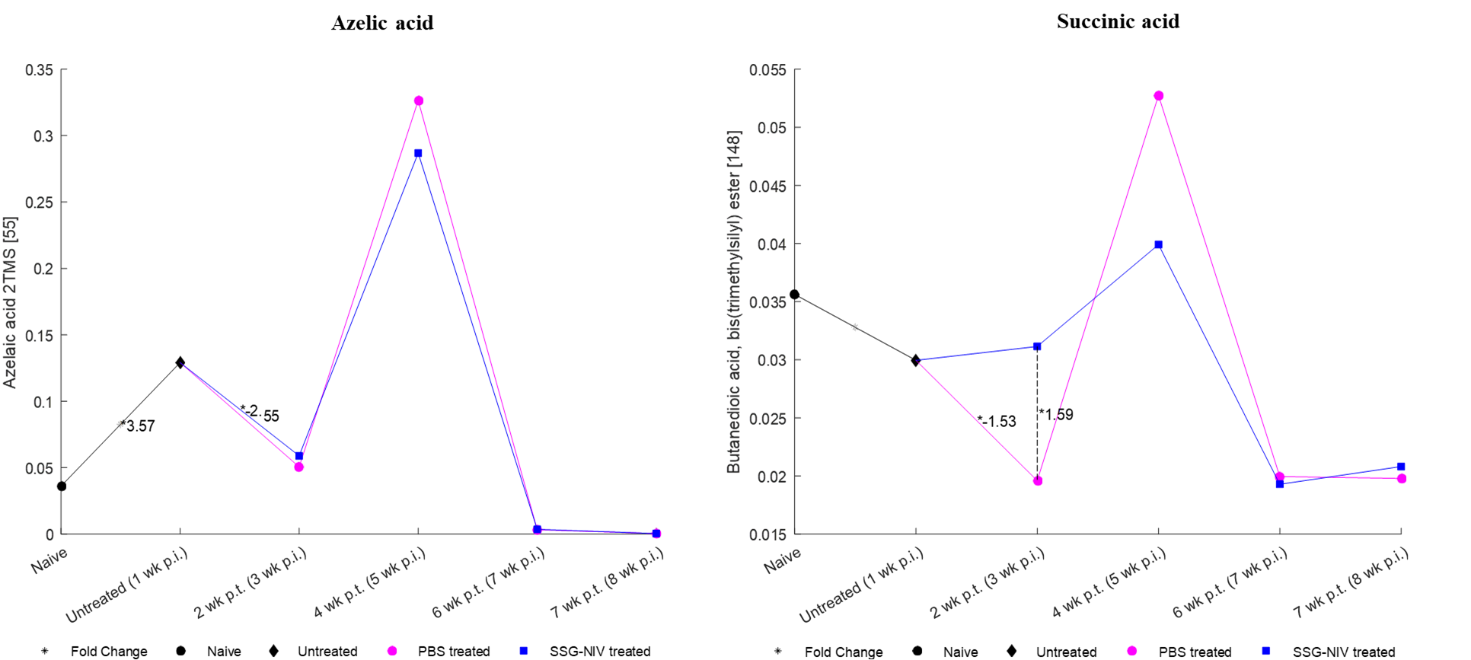


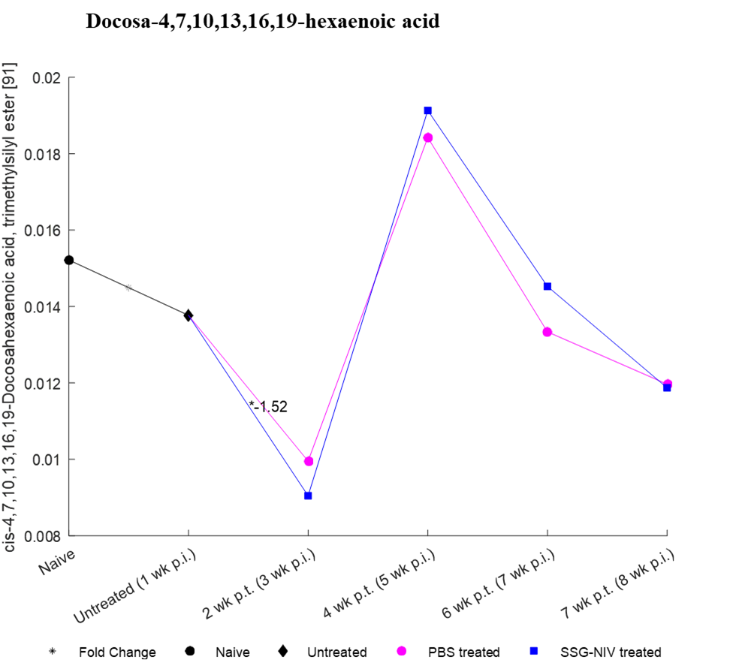

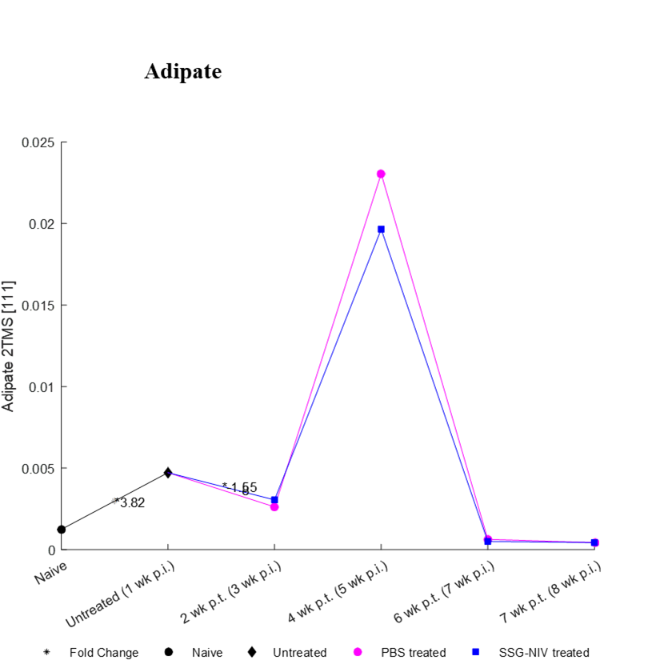


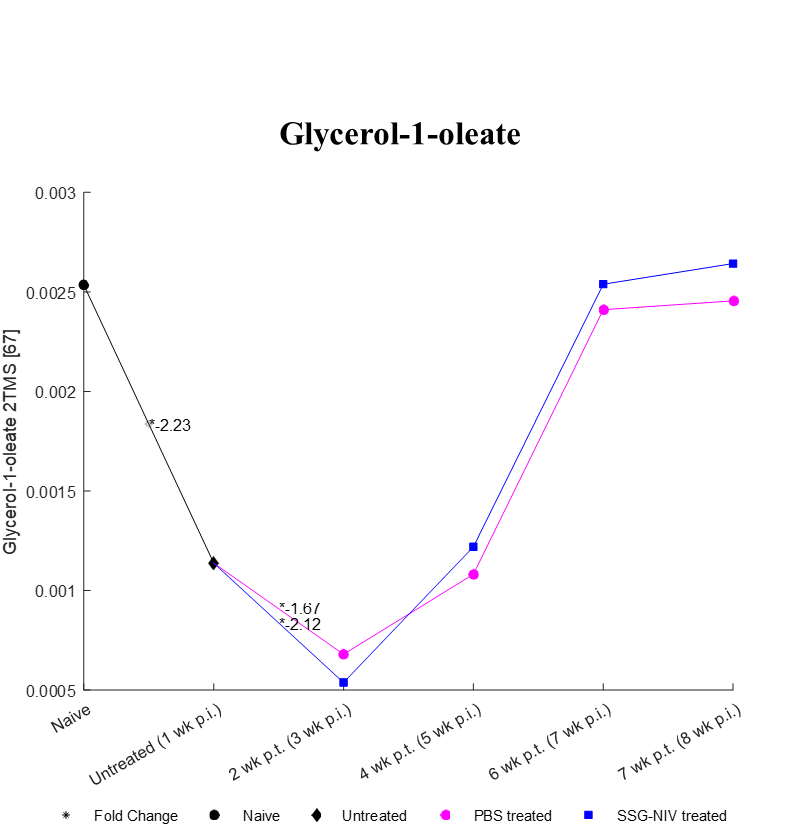


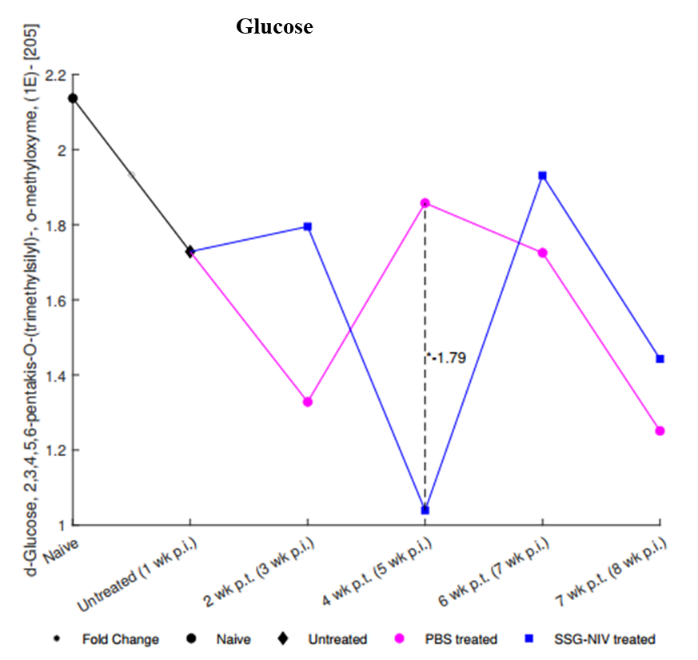


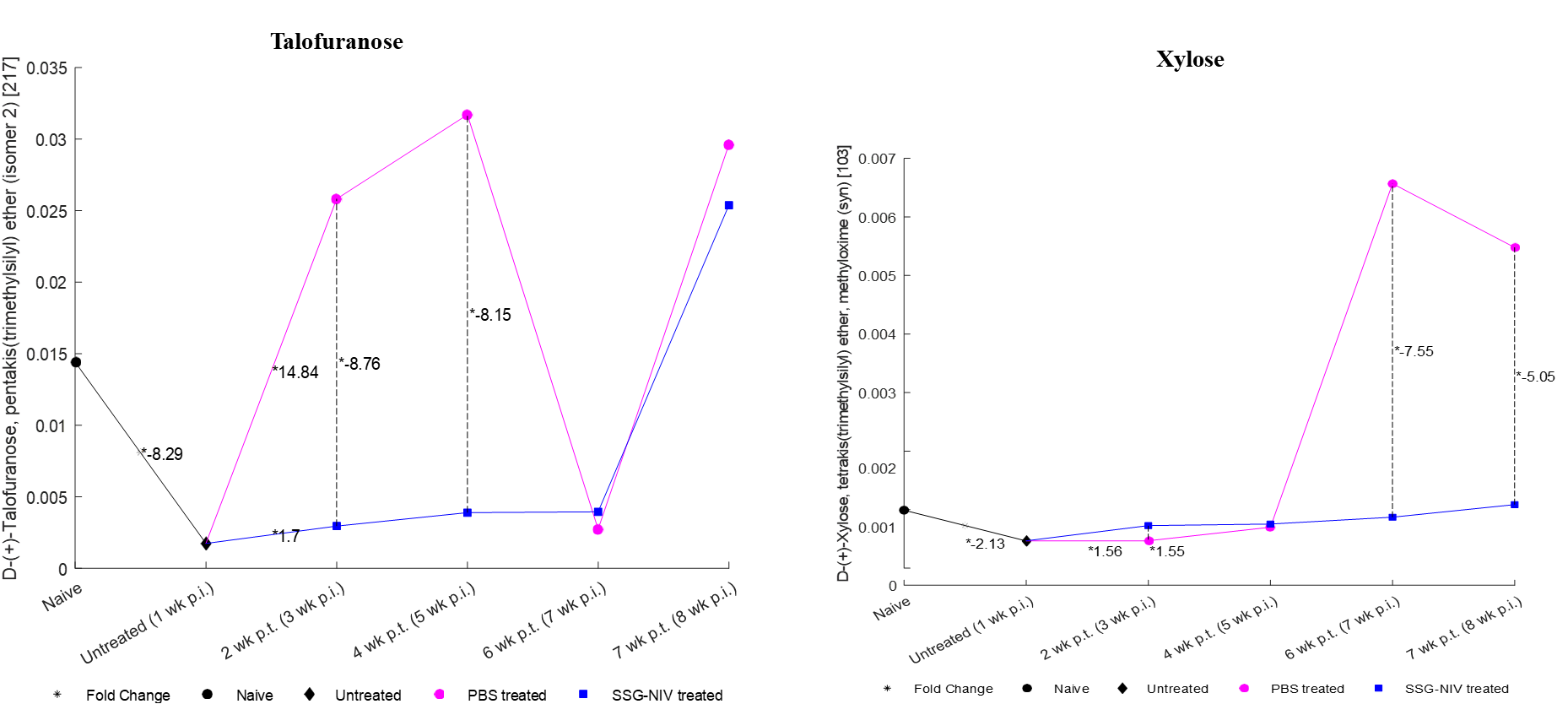


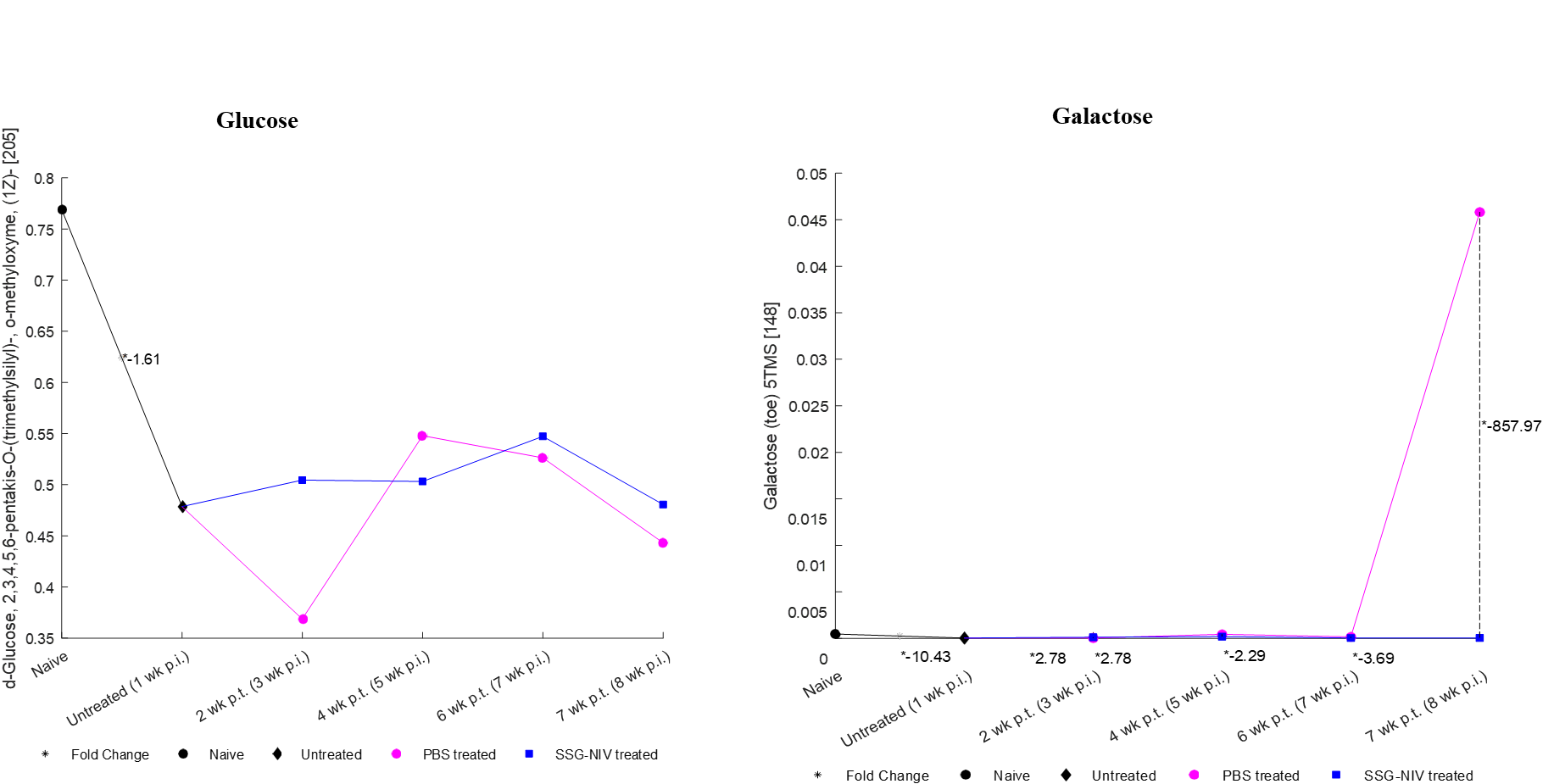


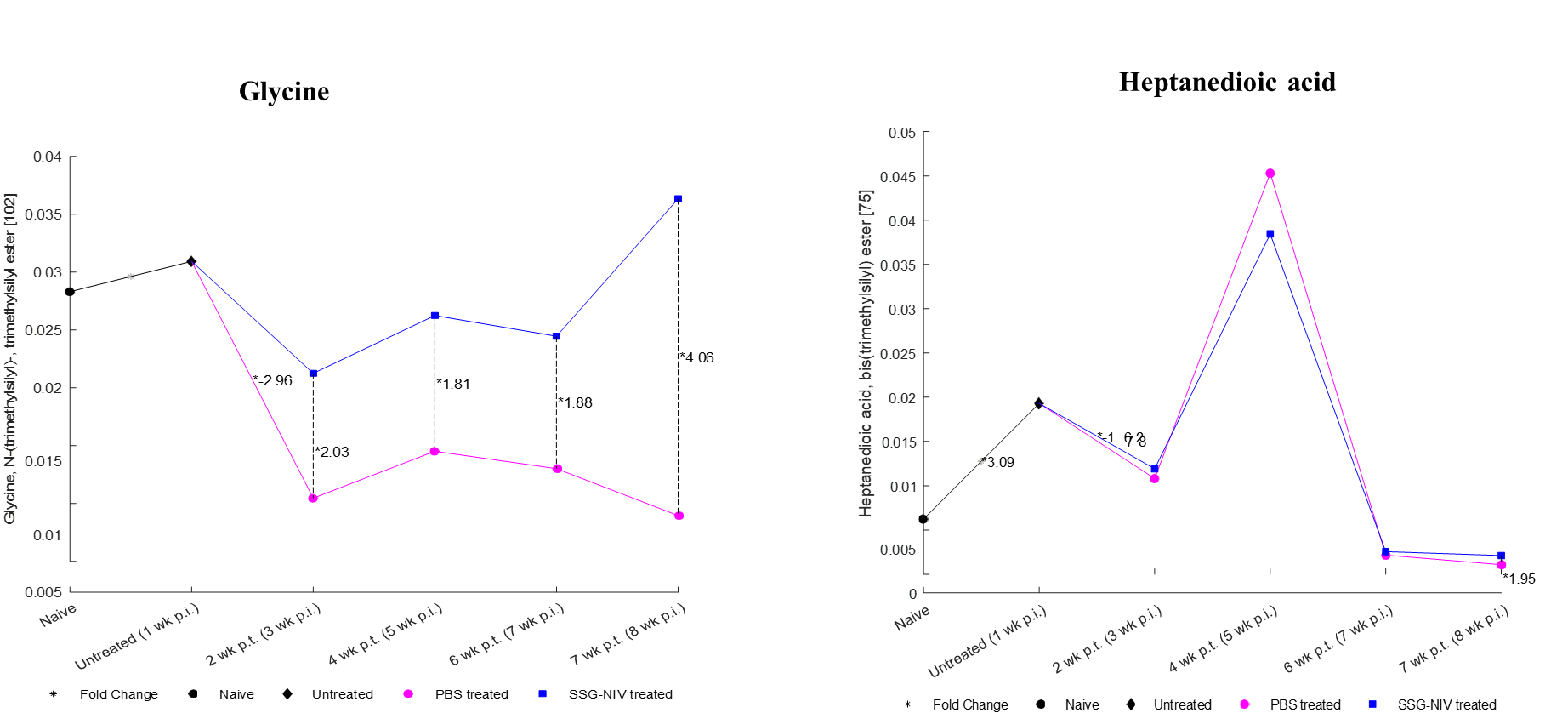


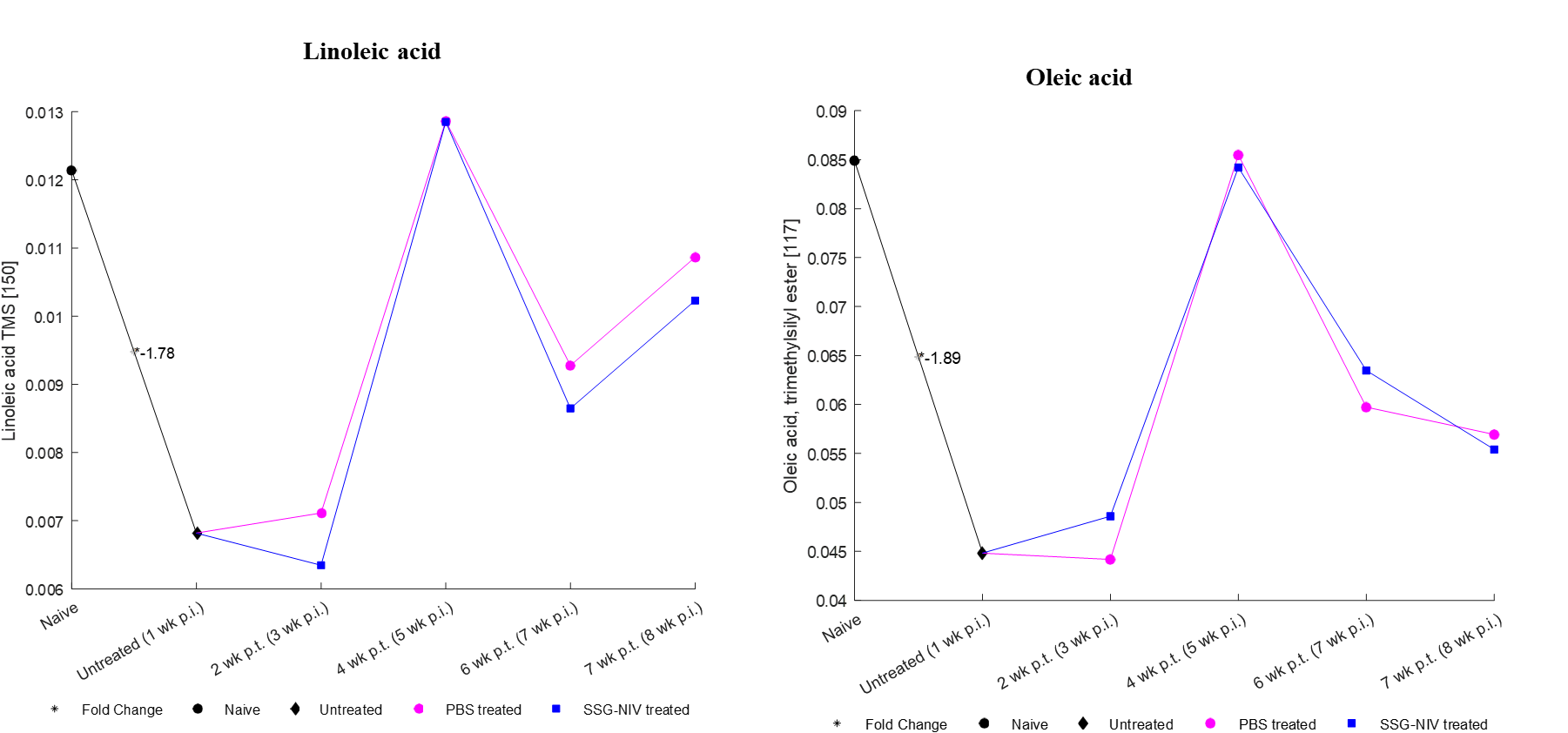


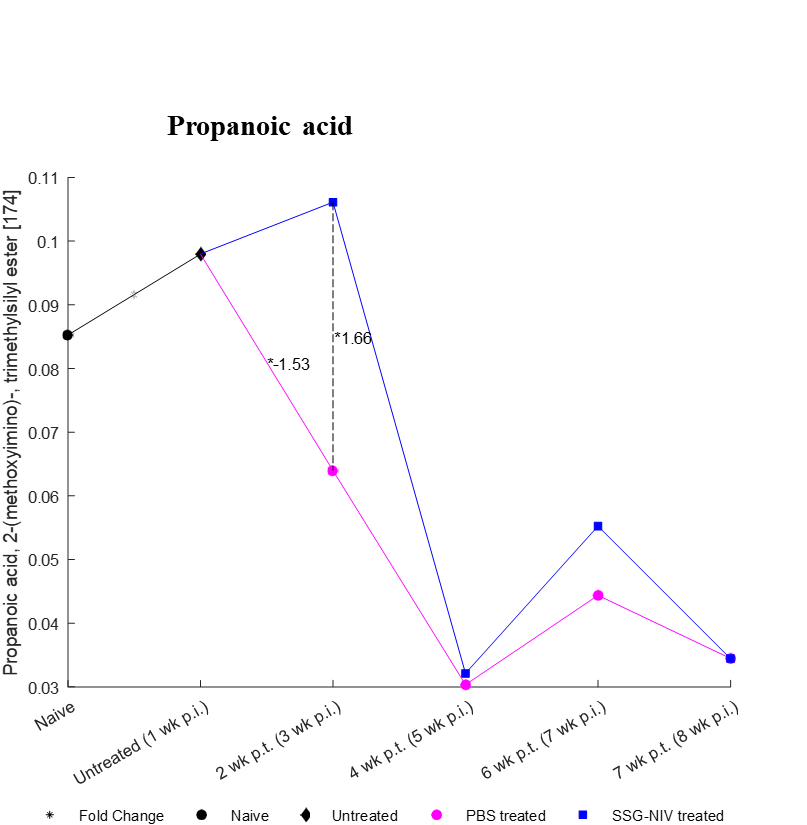

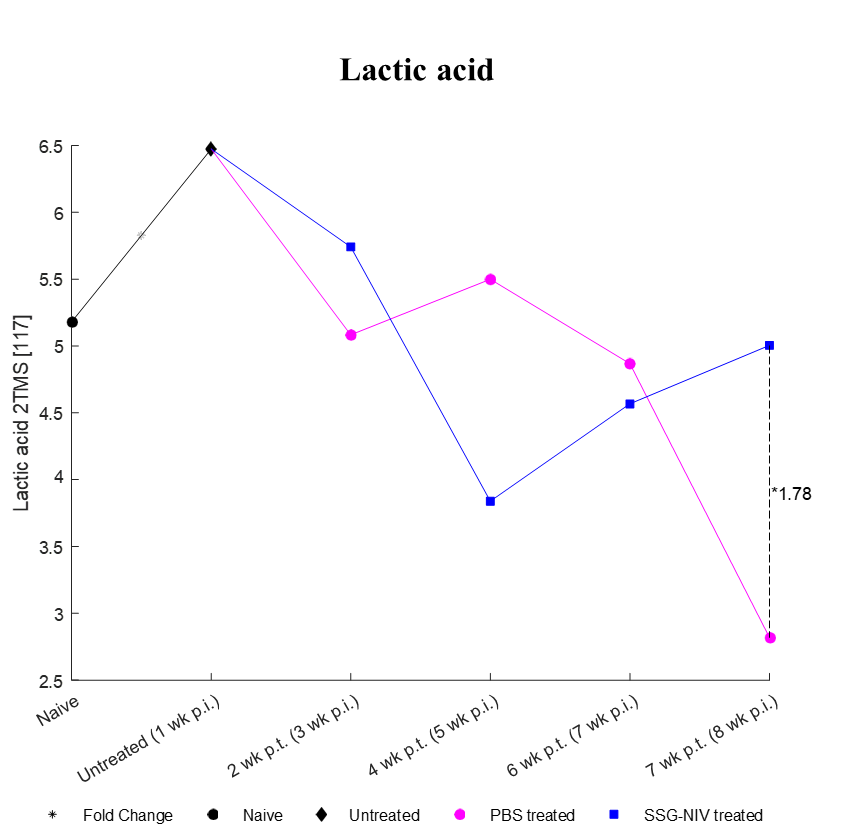


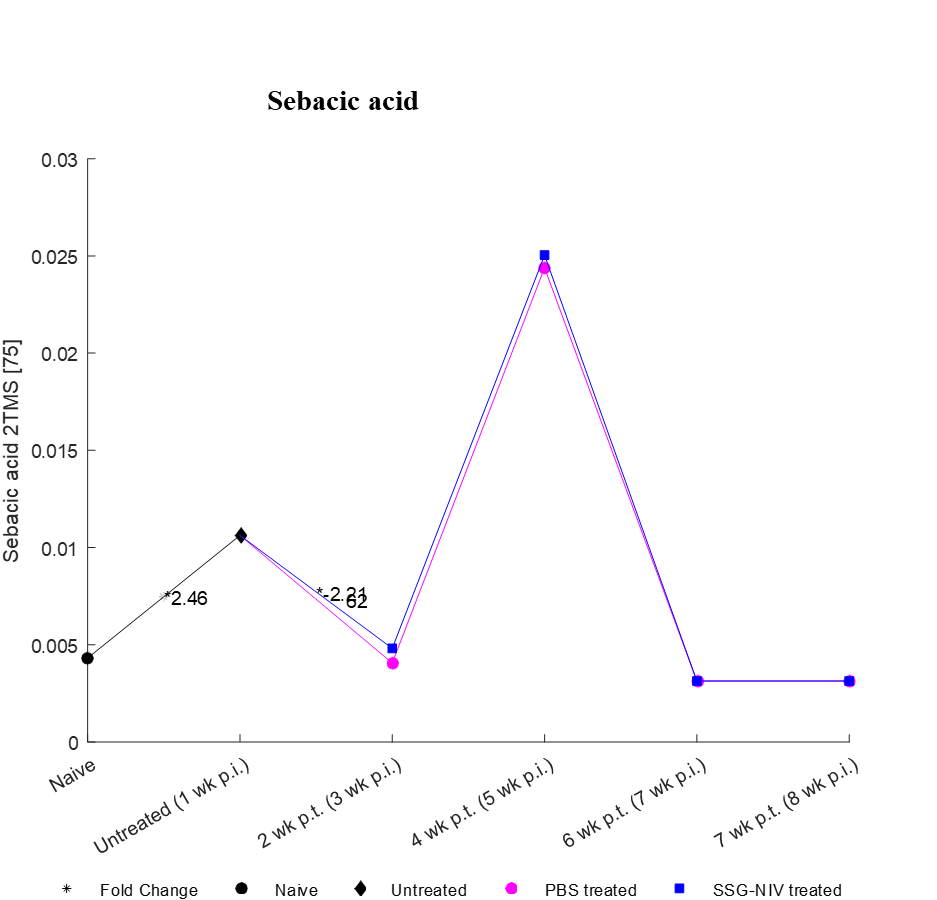

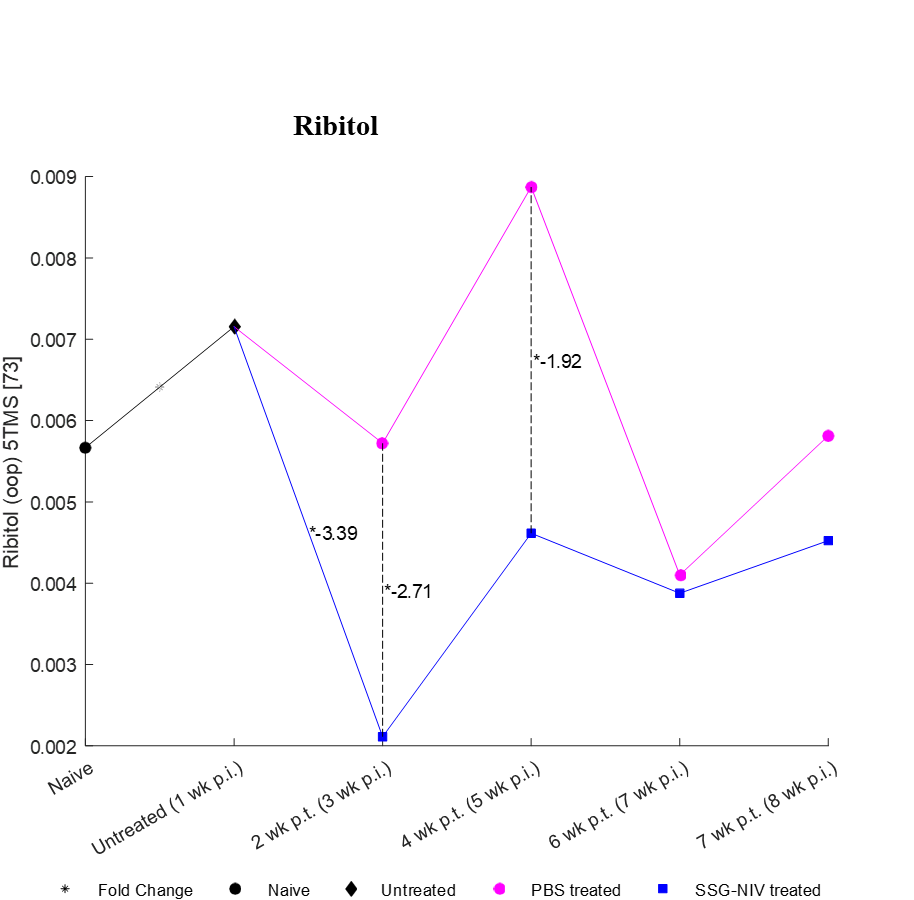


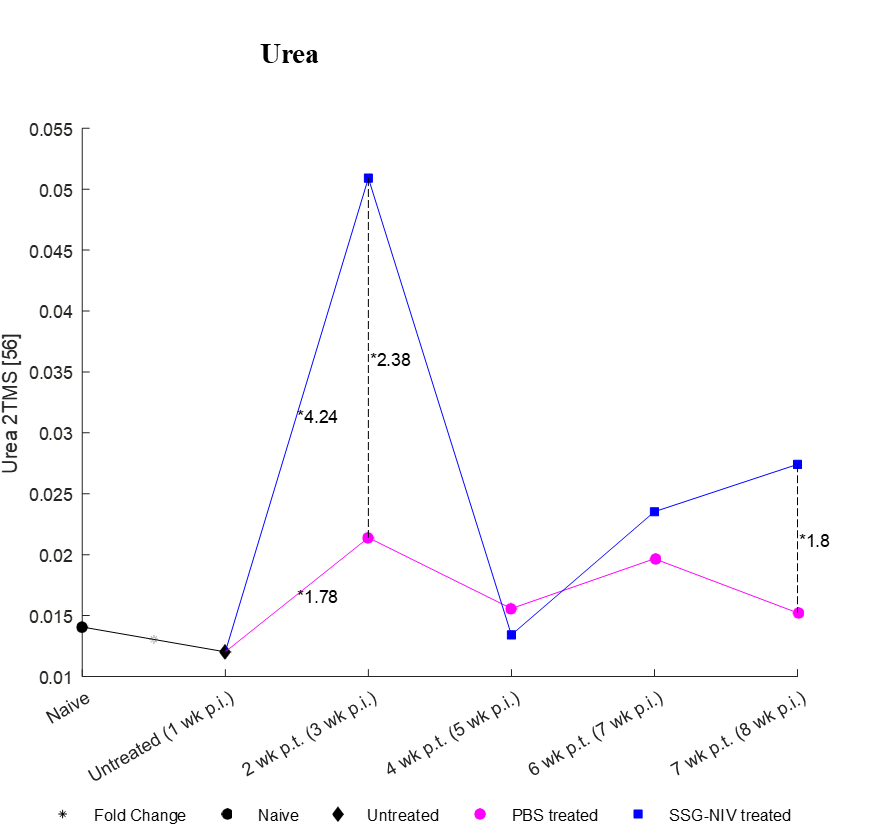

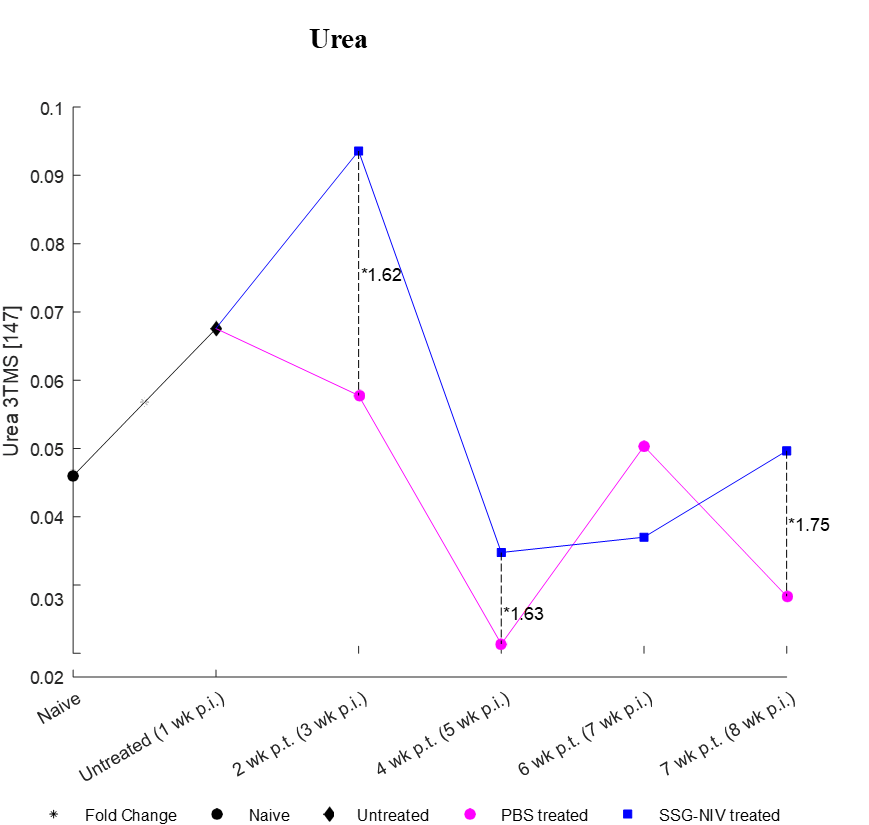


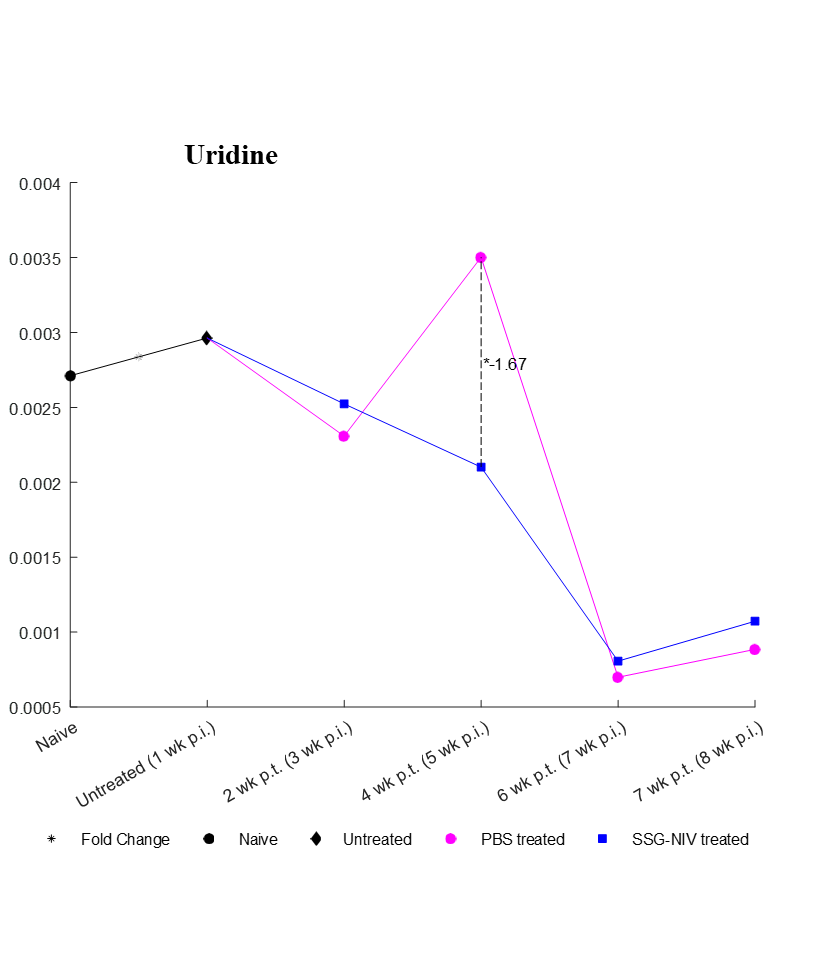


**Supplementary Figure 3:** Plots of fold change metabolites associated with treatment outcome during experimental VL. BALB/c mice were infected with 2 × 10^7^ *L. donovani* amastigotes. Infected mice were treated with either SSG-NIV (300 mg Sb^V^/kg) or PBS (controls).


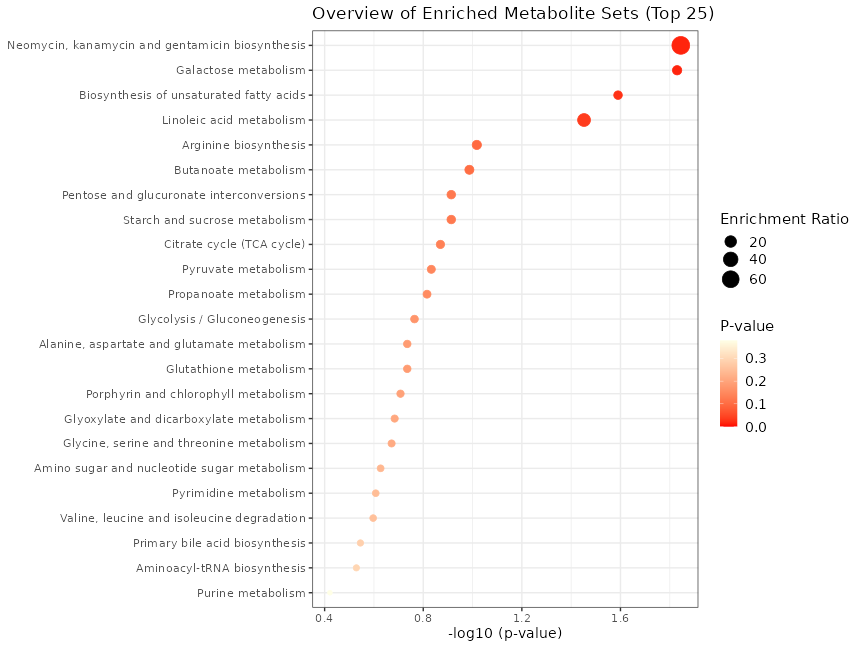


**Supplementary Figure 4. Pathway analysis of significant metabolites differentiating the SSG-NIV-treated and control (PBS) mice**. Serum samples from *L. donovani* SSG-NIV-treated and control BALB/c mice at weeks 2, 4, 6, and 7 PT were analyzed using GC×GC-TOFMS. Data analysis, conducted with MetaboAnalyst, identified significant metabolic pathways influenced by the treatment.
